# Analysis of group evolution prediction in complex networks

**DOI:** 10.1371/journal.pone.0224194

**Published:** 2019-10-29

**Authors:** Stanisław Saganowski, Piotr Bródka, Michał Koziarski, Przemysław Kazienko

**Affiliations:** 1 Department of Computational Intelligence, Faculty of Computer Science and Management, Wrocław University of Science and Technology, Wrocław, Poland; 2 Department of Electronics, Faculty of Computer Science, Electronics and Telecommunications, AGH University of Science and Technology, Kraków, Poland; Nanyang Technological University, SINGAPORE

## Abstract

In the world, in which acceptance and the identification with social communities are highly desired, the ability to predict the evolution of groups over time appears to be a vital but very complex research problem. Therefore, we propose a new, adaptable, generic, and multistage method for Group Evolution Prediction (GEP) in complex networks, that facilitates reasoning about the future states of the recently discovered groups. The precise GEP modularity enabled us to carry out extensive and versatile empirical studies on many real-world complex / social networks to analyze the impact of numerous setups and parameters like time window type and size, group detection method, evolution chain length, prediction models, etc. Additionally, many new predictive features reflecting the group state at a given time have been identified and tested. Some other research problems like enriching learning evolution chains with external data have been analyzed as well.

## Introduction

Network science is a very interdisciplinary domain focusing on understanding the relational nature of various real-world phenomena using for that purpose diverse network models. Commonly, networks consist of smaller, more integrated structures called groups, communities, or clusters. In practice, both the groups and whole networks evolve and change their profiles over time. Hence, their analysis demands advanced computational methods to understand and predict their future behavior. For that reason, group evolution prediction is an essential component of computational network science.

One of the domains explored by network science are biological networks [1–4]. Viruses are as old as life on earth. At the same time, they are very young, as they constantly mutate to change their lethal attributes. Influenza, unlike other viruses which are rather stable, evolves much more rapidly [5, 6] and kills up to one million people worldwide every year [7]. We can try to protect ourselves using vaccines. However, the rate of mutation is too rapid to provide an effective cure. What is more, the development of a new drug requires a huge amount of money and lasts from a few to a dozen or so years. Despite these difficulties, new drugs are introduced to the market every year. For example, antagonist drugs (also called blockers) are designed to bind to specific receptors to block the disease’s ability to attach to these particular receptors, thereby immunizing the body to the disease. Unfortunately, diseases react to drugs and eventually mutate, creating a variety that will bind to other receptors. Therefore, we need methods that will be able to track the evolution of the disease, and based on the history of its mutations, will be able to predict the most likely future mutations. To track diseases mutations, we can focus on the group of receptors that it binds to, and observe how such group evolves. Based on the history of changes in the lifetime of this group, we can try to predict what will be the next change. Predicting the direction of the mutation could significantly reduce the amount of time and money needed to study the disease. With such knowledge, we would be able to start preparing the drug in advance and bring it to the market much faster and cheaper.

Another area that widely applies network science, especially its branch called social network analysis (SNA), is marketing, in particular advertising [8–11]. Let us imagine that a start-up company invented a new generation of diapers—*Smart Diapers*, which are extra soft, super absorbing, and additionally, can communicate with parents’ smartphones to notify when their change time comes. The company invested very much in their development, therefore, it has a limited budget to advertise the product. The owners decided to introduce the product to discussion groups on the Facebook platform where parents from different countries/cities create and join independent groups to talk about and comment on new products for babies, share general advice about raising children, sell used clothes, etc. Convincing members (parents) of such relevant, targeted groups to use and buy the new diaper product would be much more effective and cheaper than advertising the broader community using expensive TV commercials. Additionally, the word-of-mouth recommendation is commonly believed to be the most powerful marketing tool [12]. However, the vital question rises here: which Facebook groups the company should invest in its limited resources, i.e., time and money? In the newly created relatively small groups that might be very active and are expanding fast, or in the larger groups that might be not very active in the nearest future? Which of these groups will be still running or growing in a few weeks/months/years and which one will disappear? That is why the knowledge about the history, current state, and future evolution of groups is crucial at decision making on where to allocate the resources.

In 2007, Palla et al. [13] have defined the problem of group evolution identification. In the following years, dozens of solutions to this problem have been proposed. One of them was the highly cited GED method [14]. Existing surveys describe as many as 12 [15] or even over 60 methods [16]. All of them are focused on defining possible events in the community life, hence, tracking the historical changes. This, in turn, has led to emerging a new problem—predicting future changes that will occur in the community lifetime. Some of the first methods concerning prediction of some aspects (e.g., determining lifespan) of the group evolution were: (1) Goldberg et al. [17]—they focused on predicting the lifespan of evolution for a group; (2) Qin et al. [18]—analyzed dynamic patterns to predict the future behavior of dynamic networks; and (3) Kairam et al. [19]—they investigated the possibility of prediction whether a community will grow and survive in the long term.

Note that the methods for tracking group evolution can be also utilized to other similar prediction problems, like link prediction [20], churn prediction [21], as well as to understand evolution of software (Unix operating system networks) [22] or dynamics of social groups forming at coffee breaks [23].

In 2012, we proposed a new concept, in which the historical group changes were utilized to classify the next event in the group’s lifetime [24]. In this first trial, we have used only event type and size of the group to describe its state at a given time. Over the next year, we have investigated the concept and adopted it to two methods for tracking group evolution—the GED [25] method and the SGCI method [26]. This resulted in the first method for group evolution prediction [27]. It was the predecessor of the GEP (Group Evolution Prediction) method described in this paper. Since then, a few more methods have been proposed. At the end of 2013, İlhan et al. presented their research with several new measures describing the state of the community and a new method for tracking group evolution [28]. In 2014, Takafolli et al. applied the binary approach to classifying the next change that group will undergo [29]. They used 33 measures to describe the state of the community. We have presented new results in 2015, where, apart from new measures, the influence of the length of the history used in the classification was examined [30]. Later the same year, Diakidis et al. adapted the GED method to conduct their research with 10 measures as predictive features [31]. In 2016, İlhan et al. presented new results and proposed a method to select measures, which should be the most useful as predictive features for a given data set [32]. More recently, Pavlopoulou et al. used 19 measures already validated in other works and studied whether employing the temporal features on top of the structural ones improves prediction, as well as what is the impact of using a different number of historical community states on the prediction quality [33].

Unfortunately, all of the methods proposed to this day have some drawbacks (see the Comparison with other methods section) and have been designed to solve a particular problem, hence, their application area is rather narrow. Therefore, in this paper, a new generic and comprehensive method to predict the future behavior of the groups, based on their historical structural changes as well as experienced events, is proposed, evaluated and discussed.

Some of the contributions of this work are: decomposing the group evolution prediction problem, proposing and extensively evaluating the modular method that can be applied to any dynamic network data, proposing new predictive features, performing the features’ ranking, proposing a new concept of data set enriching, initial evaluation of the transfer learning technique, an example and discussion on the concept drift problem in group evolution prediction, reviewing all proposed methods in the field.

## Methods

### Decomposition of the group evolution prediction problem

**Fig 1:**
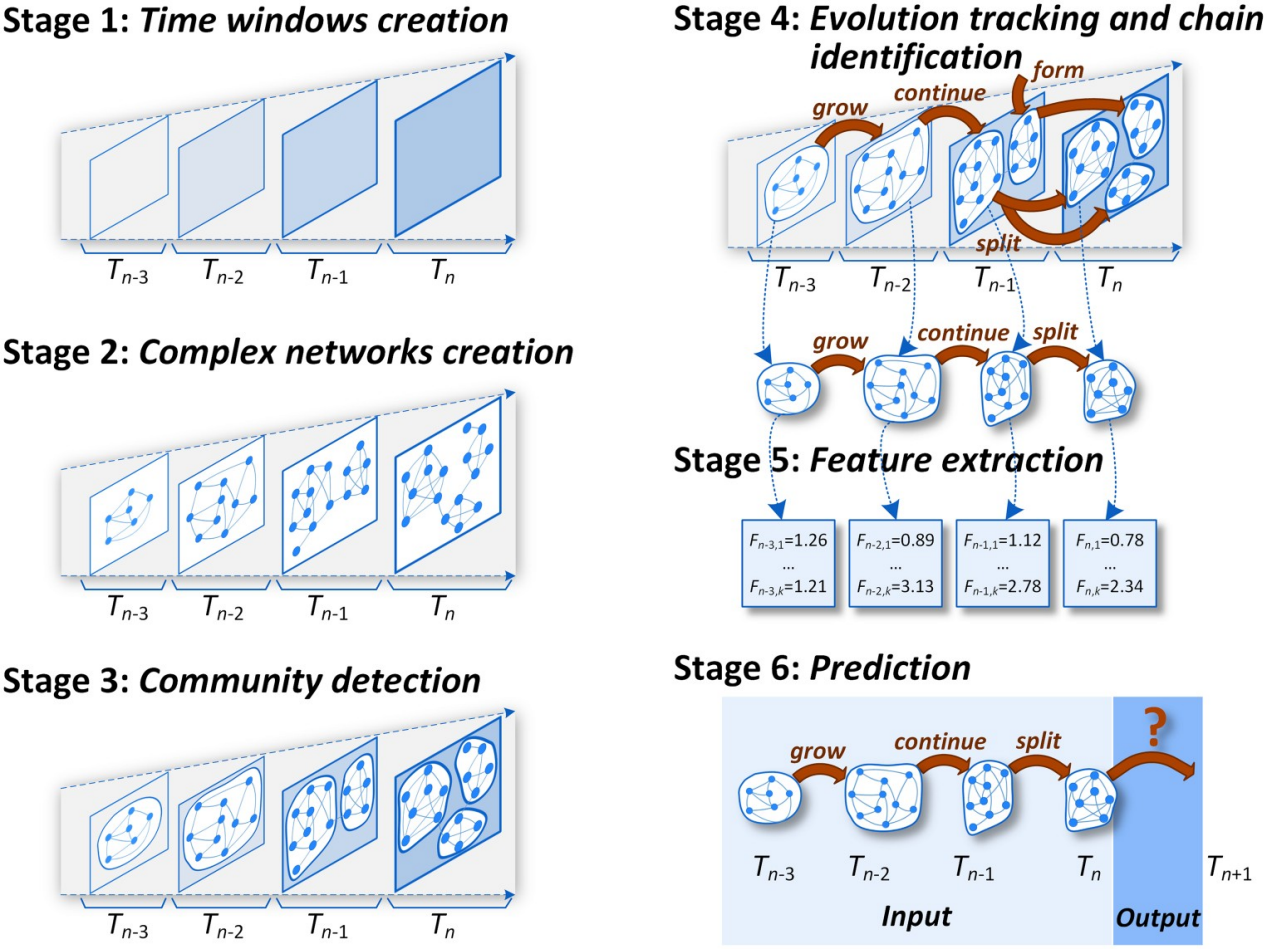
The concept of the GEP method. **Stage 1:** Data set is divided into time windows. **Stage 2:** A complex network for each time window is created. **Stage 3:** Groups are extracted within each time window using any community detection method. **Stage 4:** The evolution of communities is tracked with any group evolution tracking method, and the evolution chains are created. **Stage 5:** Features describing the previous group profile such as size, density, cohesion, etc. are calculated to capture community state at a given time. **Stage 6:** Supervised machine learning approach is applied to learn and predict the forthcoming event in the group’s lifetime.

The crucial matter in developing the modular method predicting group evolution, called *GEP*, was the identification and separation of the components of the entire group evolution prediction problem. The appropriate problem decomposition and information flow between particular components (dependencies) are depicted in Eq 1 and Fig 1.

$$\begin{array}{c} IS\overset{TWT}{\underset{S_{1}}{\to }}TW\overset{NT}{\underset{S_{2}}{\to }}TSN\overset{CDM}{\underset{S_{3}}{\to }}G\overset{CETM}{\underset{S_{4}}{\to }}EC\overset{FE}{\underset{S_{5}}{\to }}PF\overset{classification(CH)}{\underset{S_{6}}{\to }}Q \end{array}$$ (1)

The data from the input stream *IS* is divided into time windows *TW* using the time window type definition *TWT*. For each time window *TW*, a complex/social network is created using the network type definition *NT*, resulting in the temporal complex/social network *TSN*. Within each time window *TW* in *TSN*, some groups *G* are identified using a community detection method *CDM*. Next, similar and consecutive groups are matched using a community evolution tracking method *CETM*, as well as the transition is labeled with an event type out of the set of possible changes *CH*. The matched groups are combined into evolution chains *EC* that may consist of many successive changes. For each community state in *EC*, the feature extraction process *FE* is applied in order to obtain a set of predictive features *PF* describing the community state at a given time. Using features *PF* in the form of a vector representing each evolution chain *EC*, classification of possible changes *CH* is performed. The classification task (stage *S*_6_) is to learn and finally label the next change(s) in community lifetime. The output of the classification process is a set of classification quality (performance) measures *Q*, for example, F-measure, accuracy, precision, or recall. The identified components were converted into six stages *S*_1_-*S*_6_ of the GEP method, Fig 1.

### GEP method

The GEP framework consists of six main stages (Fig 1): (1) time window definition, (2) complex network extraction for the defined periods, (3) community detection in periods, (4) group evolution tracking, (5) evolution chain identification for communities together with feature extraction and computation for each chain and (6) classification, containing classification model learning and testing. Each of them can be implemented by means of different methods and approaches depending on research need and prerequisites, e.g., complexity level. The formal definition of the GEP method is as follows:

**Definition 1** *The GEP method is defined as an octuple*

< *IS*, *S*_1_, *S*_2_, *S*_3_, *S*_4_, *S*_5_, *S*_6_, *Q* >, *where:*

IS is an input stream of activities, e.g., phone calls, linking two actors (network nodes) x, y at time t_i_;

*S*_1_ *is a set of considered time windows of the given type TWT;*

*S*_2_ *is a set of considered approaches to temporal complex / social network TSN creation from IS using time window definitions from S*_1_;

*S*_3_ *is a set of considered approaches to community detection methods CDM for each time window in TSN from S*_2_;

*S*_4_ *is a set of considered approaches to tracking community evolution methods CETM for communities from S*_3_;

*S*_5_ *is a set of considered approaches to feature extraction for evolution chains from S*_4_;

*S*_6_ *is a set of considered approaches to classification, including learning, training, validating, undersampling, oversampling, and feature selection techniques;*

*Q is a set of considered classification quality measures, for example, F-measure, accuracy, precision, recall, estimated based on the classification results from S*_6_.

The methods enumerated especially in *S*_1_, *S*_3_, *S*_4_, *S*_6_ also include the space / set of their parameters.

The output of one stage *S*_*i*_ is the input for the next stage *S*_*i*+1_, e.g., communities detected in *S*_3_ are used to discover their evolution in *S*_4_. All these stages, together with parameters of the methods used, are more in-depth described in S1 File. They also require an appropriate definition of data structures to facilitate hassle-free implementation.

### CPM method

The Clique Percolation Method (CPM) proposed by Palla et al. [34] is the most widely used algorithm for extracting overlapping communities. The CPM method works locally, and its primary idea assumes that the internal edges of a group have a tendency to form cliques as a result of high density between them. Oppositely, the edges connecting different communities are unlikely to form cliques. A complete graph with *k* members is called k-clique. Two k-cliques are treated as adjoining if a number of shared members is *k*–1. Lastly, a k-clique community is the graph achieved by the union of all adjoining k-cliques. Such an assumption is made to represent the fact that it is a crucial feature of a group that its nodes can be attained through densely joint subsets of nodes.

### Infomap method

The Infomap method proposed by Rosvall and Bergstrom [35] uses the information-theoretic approach to cluster nodes within a network. It focuses on information diffusion across the graph and compression of the information flow description obtained from a random walker, which is chosen as a mean of information diffusion. Infomap changes the problem of finding the best cluster structure into finding the partition with the minimum description length of an infinite random walk. It follows the intuitive idea that if the community structure is present, the random walker will spend more time inside the community because of its higher edges density. It means that the transition to another cluster will be less likely.

### GED method

The Group Evolution Discovery (GED) method [25] is one of the best methods for tracking community evolution [36]. It uses inclusion measure to match similar communities from neighboring time windows. This measure takes into account both the quantity and quality of the group members. The quantity is reflected by the first part of the inclusion measure, i.e., what portion of the members from group *G*_1_ also belongs to group *G*_2_. The quality is expressed by the second part of the inclusion measure, namely, what contribution of important members from group *G*_1_ is in *G*_2_. It provides a balance between the groups that contain many of the less important members and groups with only few but key members. The inclusion measure and the group size determine the type of community change. The authors defined seven possible event types: forming, dissolving, continuing, growing, shrinking, merging, and splitting. The method can work with any community detection method and with any group similarity measure, thus, providing great flexibility.

### İlhan et al. method

The İlhan et al. method [32] works with the disjoint type of communities and utilizes the function by Hopcroft et al. [37] to calculate the similarity between two communities. The event types that can occur in the community lifetime and also the classes being classified are: survive, growth, shrink, merge, split, and dissolve. The measures used as predictive features are divided into two categories: structural and temporal community measures. In total, nine features per timeframe are used, i.e., number of nodes and edges, intra and inter measure of community edges, betweenness, degree, conductance, aging, and activeness. If one calculates four network measures beforehand (average path length, betweenness, clustering coefficient, embeddedness), the method can also identify features that should be the most prominent for a given network profile.

## Results

Suitable decomposing the problem of group evolution prediction (see the Methods section and Fig 1) was crucial in solving the problem. It allowed to analyze distinct phases of the process and to propose multiple solutions for each phase. The GEP method was extensively analyzed on fifteen real-world data sets (see S1 File for their profiles), for which more than 1,000 different temporal networks were created, and in total, more than 5,000,000 individual classification tasks were performed. However, to keep the article clear and concise, only selected results are presented for each stage.

### Stage 1: Time windows creation

At first, the data is divided into time windows. Three main approaches can be considered in this context: (1) equal length periods—the events and relations are segmented based on their timestamp; (2) the same number of relations in each time window; (3) the arbitrary division, based on the data context. Additionally, the type and size of time windows have to be decided, which may be a challenging task. There are three most common types of time windows: disjoint, overlapping, and increasing.

A proper choice of the time window type and size has a direct impact on the following GEP stages, especially on the number of evolution chains discovered by the tracking method (Stage 4). If relations between individuals in a data set have a tendency to change rapidly, then disjoint time windows would be a poor choice since there may not be too many relations lasting between two consecutive time windows. As a result, the tracking method will not provide any events (Stage 4), so there will be no input to a classifier resulting in no event to predict (Stage 6). The too large size of the time window, in turn, might lose some information about community changes that occurred in the meantime.

So far, there is no formula which determines the right type and size of the time window, but a few guidelines can be provided based on our extensive experiments:

- If the network is sparse or changes rapidly, the overlapping time window should be used. Usually, the offset equal to 30% of the time window size is enough to obtain a reasonable number of events between the consecutive time windows;
- The time window type and size should be adjusted to the context of the given data set, e.g., the co-authorship network, referring to researchers who often publish only once a year, should evolve smoothly with the 1-year disjoint time windows;
- If the persistent groups are the goal of analyses, the increasing time window should be utilized, as it provides mostly the continuing and growing events;
- If relations between individual nodes are recurrent and the network is rather dense, one may try using disjoint time windows to lower the computational cost;
- It is acceptable and even preferable to repeat the selection of the time window type and size several times to see which approach yields the best results.

The most common choice in our studies was the overlapping time windows with the offset between 30%–50% of their size.

### Stage 2: Formation of networks

The parameters that can be adjusted at the creation of networks for each time window is the set of edge attributes, in particular, their weights and direction. The weighted/unweighted, as well as directed/undirected profile of the network, did not yield a significant impact on computational complexity nor classification accuracy. Some community detection methods, however, may be incompatible with the networks of particular characteristics or may ignore some attributes, e.g., weights. The CPM [34] and Infomap [35] methods, used in the experimental studies, are capable of handling the most important network attributes.

### Stage 3: Community detection

**Fig 2:**
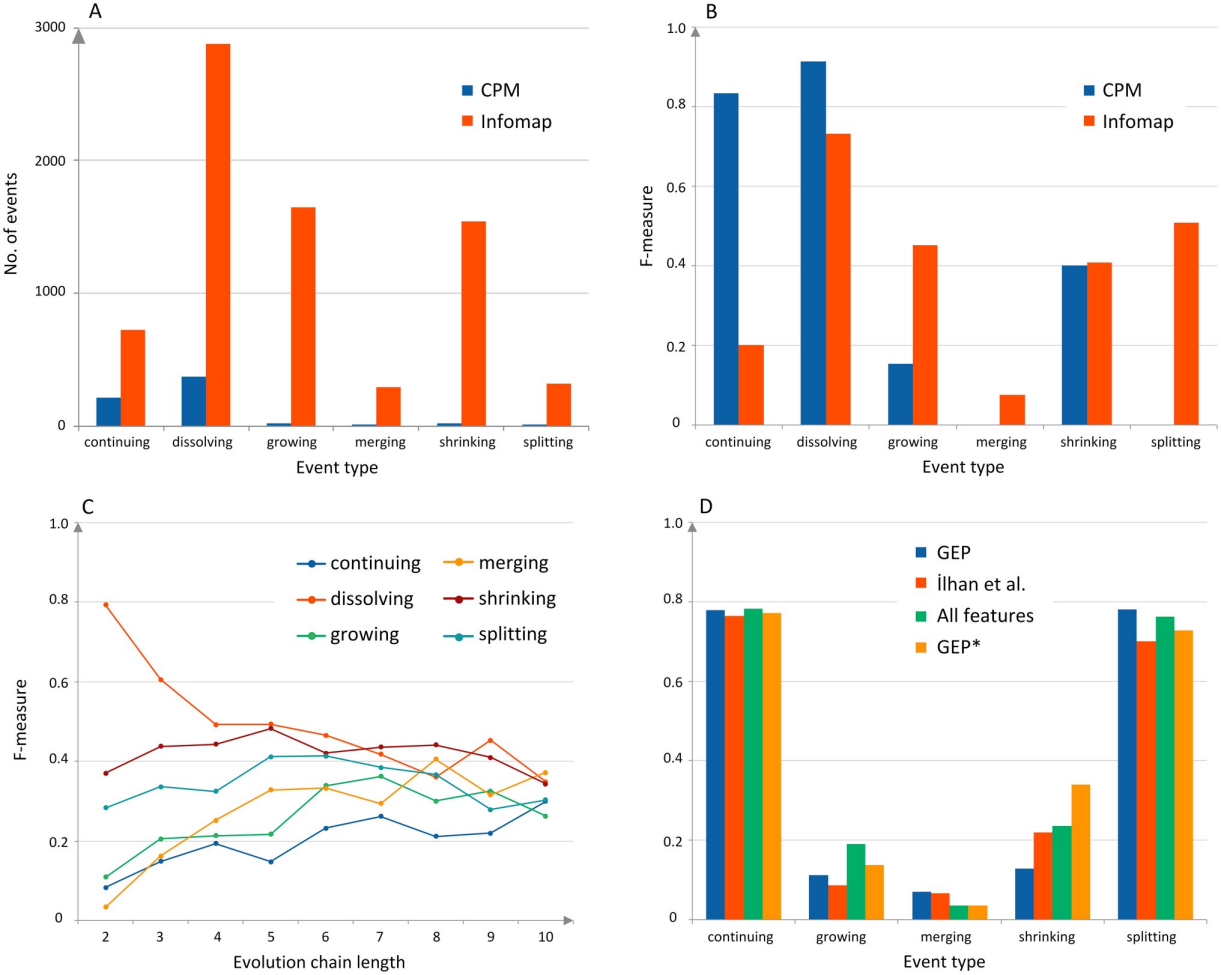
**(A) CPM vs. Infomap**. The number of events tracked with the GED method for groups obtained with two different community detection methods applied to the Digg data set. The CPM method leaves out even 97% of nodes that do not belong to any clique, hence the small number of groups and events. **(B) CPM vs. Infomap**. The F-measure values achieved for the events presented in Fig 2A. The results reflect the distribution of events. **(C) Chain length**. The F-measure values for different lengths of the evolution chains for the Facebook data set. For most of the events, the F-measure value was increasing with the increase of the chain length up to 6 or even 7 states (the continuing and growing events). Beyond that point, the number of evolution chains of the particular types dropped below 50 which was insufficient to train the classifier properly; **(D) GEP vs. Ilhan et al**. The F-measure values for the 9-state evolution chains obtained from the Slashdot data set with the different set of predictive features: only from the GEP method (GEP)—see S1 File, from the İlhan et al. method, combined from both GEP and İlhan et al. methods (All features), and from the GEP method, but only for the last 3 states out of all 9 states (GEP*). The GEP* and “All features” scenarios achieved slightly better overall scores.

Some community detection methods can produce both disjoint and overlapping communities, but there are only a few methods for tracking the evolution (Stage 4) that can deal with the overlapping groups. Overall, the methods extracting disjoint communities perform faster than the ones providing overlapping groups. In some extreme cases, when the network is very large, the CPM method is unable to extract groups due to its enormous memory requirements. It is hard to compare two types of the grouping methods in terms of their impact on the classification accuracy, as each type of clustering delivers a different set of communities resulting in a different distribution of evolution events. Besides, the profile of the groups may be diverse, e.g., networks grouped with the CPM method tend to have a single giant component with many small overlapping groups alongside. This method also inclines to leave out nodes that do not belong to any clique, thus, excluding them from further consideration. If the network is sparse, a major fraction of the network may be omitted. In the most extreme case, the CPM method neglected even as many as 97% of network nodes, what resulted in a deficient number of communities and evolutions (Fig 2A), and eventually in very low classification accuracy, Fig 2B. At the same time, the Infomap method performed very well, identifying a large number of communities. Furthermore, the overlapping groups are likely to generate more merging and splitting events in Stage 4, since there are plenty of similar and overlapping communities in the consecutive time windows. On the other hand, the Infomap method tends to produce many communities having only 2 or 3 nodes. In general, while considering which type of grouping method to use the data context should be a crucial factor.

### Stage 4: Stepwise evolution tracking and chain identification

Regardless of the method, tracking the evolution of community is a computationally demanding task. The method has to iterate over all time windows and compare all the communities in order to detect similar ones. Although the methods for tracking group evolution can be very distinct, especially while defining the possible event types, our earlier study showed that the selection of the method has no significant impact on classification accuracy [30]. In this evaluation, we use the GED method [25] since, in the last evaluation of existing community evolution tracking method, it was selected as the one giving the most satisfying results [36].

The parameters of the selected method might influence the classification results, e.g., the alpha and beta parameters of the GED method have a direct impact on the number of evolution events discovered—the lower the threshold, the more events obtained (see S1 File for details). In the experimental studies, the most common value for the alpha and beta parameters was 50%. If the network is dense and relations are recurrent, the alpha and beta might be even increased to 70%. On the other hand, when the method provides a small number of the evolution events, the alpha and beta should be reduced to, e.g., 30%. Apart from the selection of the evolution tracking method, the length of the evolution chain has to be decided. The longer the evolution chain, the more predictive features for the classifier in Stage 6, hence, the higher computational complexity. Nevertheless, the results presented in Fig 2C revealed that it is worth dedicating some more time and resources to extract longer chains since it can boost classification accuracy. The overall score achieved with the evolution chains containing six community states was 32% higher than the results achieved with shorter 2-state chains. In case of limited time or resources, the chains with the length of 2-3 states should be reasonably good.

### Stage 5: Feature extraction

**Fig 3:**
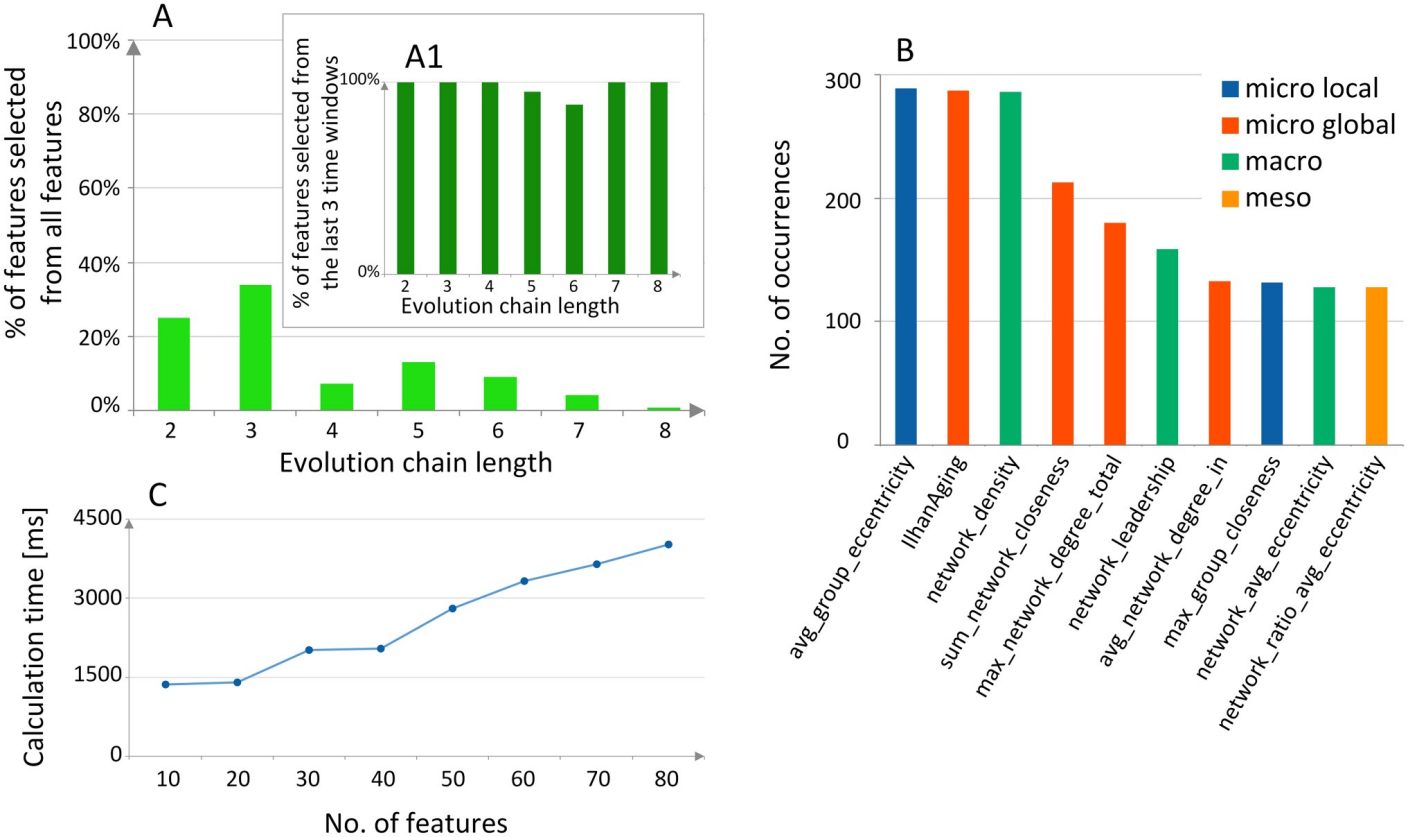
**(A) Feature selection**. Important features selection obtained by the Backward Feature Elimination for the DBLP data set. The total number of features increases with every state by 91, e.g., the 3-state evolution chain has 91*3 = 273 features in total, out of which 34% were selected as prominent. **(A1)** Features selected only from those related to the last 3 time windows. **(B) Feature ranking**. The most frequently selected features for the 1-state evolution chains. All kinds of information are important to achieve a satisfactory prediction; microscopic features are focused on nodes, mesoscopic on groups, and macroscopic on entire network parameters. The ranking obtained by analyzing eight data sets and repeating feature selection 1000 times. **(C) Computational efficiency**. The time required to train a single Random Forest classifier in relation to the number of descriptive features used as the input data. The results obtained for the IrvineMessages data set.

In order to predict the future evolution of the group, we need to describe its recent and historical states by means of predictive features. Based on these features and previous evolutionary changes used to learn the model, we are able to forecast the next changes. The crucial features that are at our disposal are structural network measures computed for the previous group states. Calculation of all measures may be a very demanding task since they need to be evaluated for every community state in the evolution chain. Additionally, some measures, e.g., betweenness centrality, require finding all shortest paths for each pair of nodes in the community or network. The experiments revealed, Figs 2D and 3 that the set of predictive features has a significant impact on classification accuracy, as they are used to build the classification model, see also S1 File, Feature Selection section. Therefore, it is highly recommended to compute as many predictive features as possible to deliver to the classifier a wide variety of descriptions to choose from.

To significantly enhance the already existing approaches, many new predictive features are proposed in this paper (see S1 File, Predictive Features section). We have clustered structural features into three general types: (1) *microscopic*—calculated for individual nodes, e.g., node degree, (2) *mesoscopic*—quantifying single groups, e.g., group size—no. of nodes, and (3) *macroscopic*—describing the whole network, e.g., network density. Mesoscopic features also include normalized group measures like the group size divided by the network size. Besides, node-based (microscopic) measures can be aggregated (usually averaged) at either *local* (group) or *global* (network) level resulting in *microscopic local* or *microscopic global* features, respectively.

All computed features were thoroughly evaluated in terms of usefulness for the classifier and rankings of the most prominent features were built, see S1 File, Feature Selection section, especially Table E-I. For the evolution chains of a variable length, different rankings were obtained. For the shortest 1-state evolution chains, only macroscopic (network) features were helpful, which may result from the fact that communities with a short history are considered unstable and vulnerable to the environment they are a part of. For the evolution chains with the increasing time windows, the features describing the local structure, especially the centrality- and distance-based measures, were more informative for the classifier, as the changes between the consecutive increasing time windows were delicate and occurred at the microscopic rather than macroscopic level. The neighborhood-based features were among the most valuable features for the longest 8- and 9-state chains, which lead to believe that for the long-lasting communities, the relations with their surroundings are a better predictor of the forthcoming change than, e.g., the macroscopic features. In general, the variations of the eigenvector-, eccentricity-, and closeness-based features were present in most of the selective rankings, which suggests that centrality- and distance-based measures obtained on the node level are the most prominent ones. Hence, in case of limited computational capacity, these features should be respected before any other. However, out of all features considered by the classifiers, the Backward Feature Elimination selected only up to 34% of them as prominent, i.e., used by the classifier to make a decision, Fig 3A.

Additionally, it turned out that usually over 90% of the selected prominent features were obtained from the last three community states, Fig 3A1. For example, when the evolution chain length was 8, and the next change was classified, all the prominent features were from the 8th, 7th, and 6th group profiles. It means that the most recent history of the community has the most significant impact on its next change. This is an extremely useful conclusion if one has limited computational capabilities and cannot calculate community profiles for all states or does not possess data about older history. The number of features has a direct impact on the duration of the entire learning process, Fig 3C.

### Stage 6: Prediction

In the last stage, the machine learning techniques, such as oversampling, undersampling, feature selection, and first of all, model training and adjustment are applied to achieve the highest possible prediction quality. The common problem with the training data is an imbalanced distribution of output classes, Fig 2A. In extreme cases, when one class greatly dominates over the other ones, a trained model tends to assign the dominant class to most observations. Then, the solution is to apply additional preprocessing techniques like oversampling and undersampling to generate additional observations or to filter out predominant ones, thus providing a distribution closer to flat. Another common problem is overfitting the classifier by providing too many features or observations. In order to prevent from such case, feature elimination technique may be applied, which unfortunately is very expensive in terms of computational complexity.

**Fig 4:**
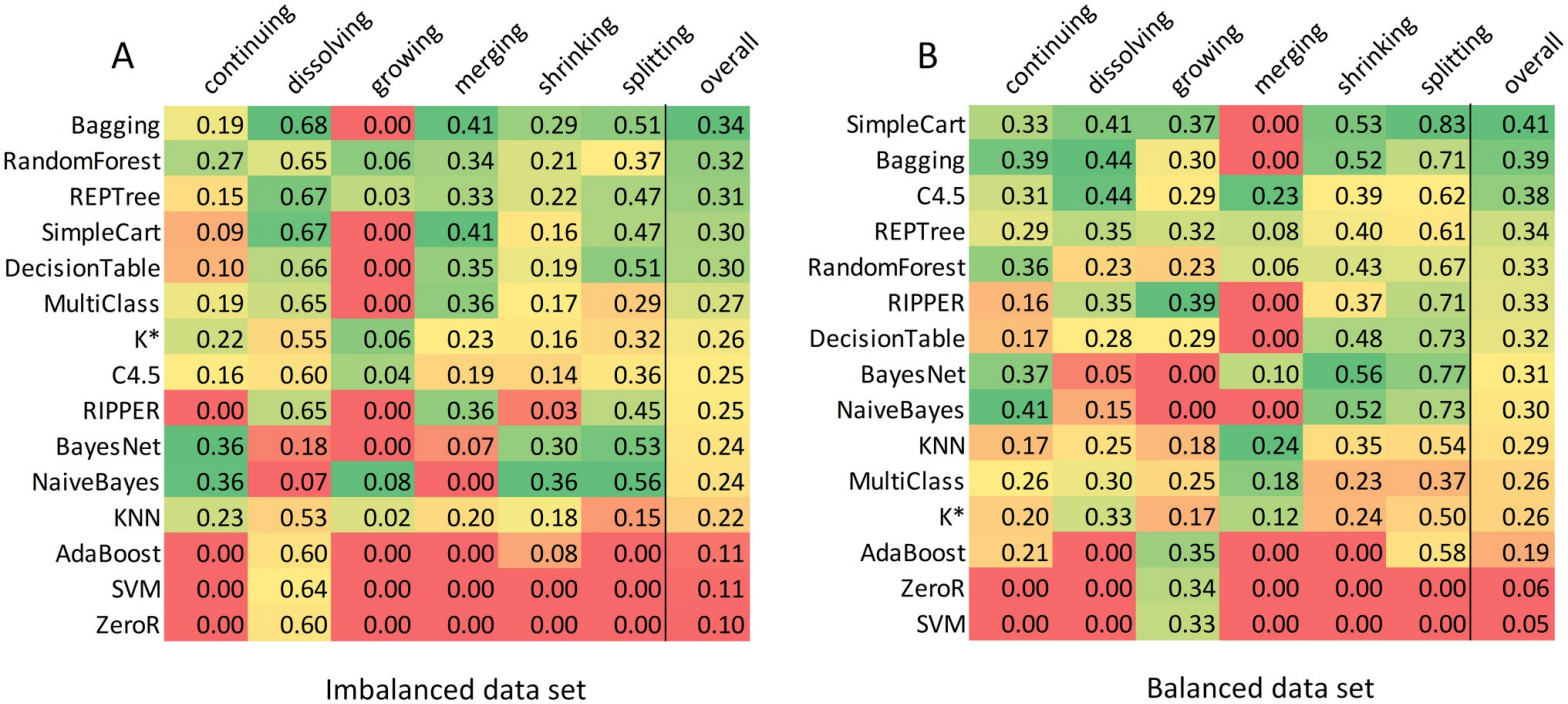
The rankings of classifiers. The heat-maps of the F-measure results for the 1-state evolution chains obtained from the Twitter data set. Classifiers are ordered by the overall score. The Bagging classifier and the SimpleCart classifier achieved the highest overall scores but failed to predict the growing and the merging events. Therefore, the tree-based classifiers are the best choice as all the events are successfully classified and the overall score is insignificantly lower.

Additionally, the proper classifier should be selected, and its parameters need to be accordingly adjusted. In the experimental study, fifteen different classifiers were compared in terms of the classification accuracy, Fig 4. The tree-based classifiers and meta-classifiers (equipped with decision trees) performed best. Many classifiers could not efficiently handle imbalanced data, so the undersampling and oversampling techniques were applied, resulting in notably better prediction quality, Fig 4B. On the balanced data set, a classifier focuses on the predictive features computed for the community states instead of focusing on the event distribution.

The Friedman statistical test [38] with the Shaffer post-hoc multiple comparisons [39] was performed to obtain rankings of classifiers on the imbalanced and balanced data sets (cf. S1 File, Table J). In both cases, the Bagging classifier (with the REPTree classifier) was the winner, and the Random Forest classifier was ranked second. What is essential, the p-values confirmed that the results were statistically significant.

Furthermore, classifiers often have their parameters to tune them accordingly, which can substantially affect the classification accuracy, cf. S1 File for detailed discussion. For example, the logarithmic correlations were observed between the number of bagging iterations for the Bagging classifier and the average F-measure value, as well as between F-measure and the number of generated trees by the Random Forest classifier. The results prove that the process of adjusting the classifier parameters should always be performed, as long as the computational time and resources are available.

### Comparison with other methods

The GEP method was compared to other approaches. The existing methods for group evolution prediction were additionally analyzed, and many of their drawbacks have been identified. The most severe were: a narrow application area, methodological issues (e.g., inappropriate computation of the conditional probability), insufficient validation of the methods (e.g., a single sampling into two folds instead of the 10-fold cross-validation), superficial descriptions of the methods and conducted experiments (often insufficient to repeat and validate the experiments), and lack or unreliable comparisons with other methods.

Despite GEP is so flexible and has so many options, it is competitive with other approaches, designed to deal with a specific problem or data set. For example, a special version of the GEP method, in which only features from the last three states (out of all 8 or 9 states) were used as an input for the classifier, performed noticeably better than the method by İlhan et al. [32], Fig 2D.

After all, it needs to be emphasized that none of the existing methods is as adjustable and versatile as the GEP method.

## Discussion

Across its six stages, the GEP method utilizes various approaches, methods, and techniques, which can be adjusted with respect to a given data set and a particular study purpose. These approaches, methods, and techniques are considered as the GEP method parameters. To provide a concise summary of their impact on overall computational complexity, and first of all on the final classification accuracy, the crucial parameters were listed in Table 1 and discussed throughout the article.

**Table 1:** The GEP method parameters and their impact on computational complexity and classification accuracy.

Parameter group	Parameter	Parameter value	Impact on computational complexity	Impact on classification accuracy
time window	window division	timestamp / relations count / arbitrary	none	low
	window size	time unit or number of relations	medium	low
	window type	disjoint / overlapping / increasing	medium	medium
network type	edge attributes	directed / undirected, weighted / unweighted	low	low
methods	group type	disjoint / overlapping	medium	low
	grouping method	a method	high	medium
	tracking method	a method	medium	low
	GED alpha and beta	(10%, 100%]	none	low
	GED social position measure	a measure	medium	low
classification	classifier used	a classifier	medium	medium
	machine learning techniques	undersampling, oversampling, feature selection	high	high
other	evolution chain length	number of community states	medium	medium
	predictive features	a set of features	high	high

Many different classifiers were evaluated on various data sets. The tree-based classifiers and meta-classifiers (equipped with decision trees) performed best. Many classifiers could not handle imbalanced data sets, so the undersampling and oversampling techniques were applied. Balancing data sets notably improved the results confirming the usefulness of the undersampling and oversampling methods. The experimental studies showed that adjusting the classifier parameters can significantly improve classification accuracy. The logarithmic correlations were observed between the number of bagging iterations in Bagging classifier and the average F-measure value, as well as between the number of generated trees by the RandomForest classifier and the average F-measure value. The confidence factor parameter of the J48 classifier was found also correlated with the average F-measure value. The maximum improvement in average F-measure value achieved by adjusting the classifier parameter was 17%, and it was obtained by increasing the number of generated trees by the RandomForest classifier. The results prove that the process of adjusting the classifier parameters should always be performed, as long as the computational time and resources are not limited.

The GEP method enables us to consider different new scenarios, which are hardly available without this generative framework like transfer learning, class balancing by adding external data, or decreasing the concept drift effect.

The transfer learning technique was adapted to the problem of group evolution prediction for the first time in this field. Its main idea is to learn the classification model on one data set and test it on another one. Such an attempt was quite successful, and the preliminary results were satisfactory. The key to success is finding a data set with a likewise profile. Moreover, in some cases, learning the transferred model on the balanced data set can boost the classification quality for the data set to which the model is adapted. The initial experiments also suggest that the underlying similarity of two data sets (e.g., the same habits of actors or ideally the same set of actors) can help to create a model that if transferred can outperform the primary model built for a given data set.

**Fig 5:**
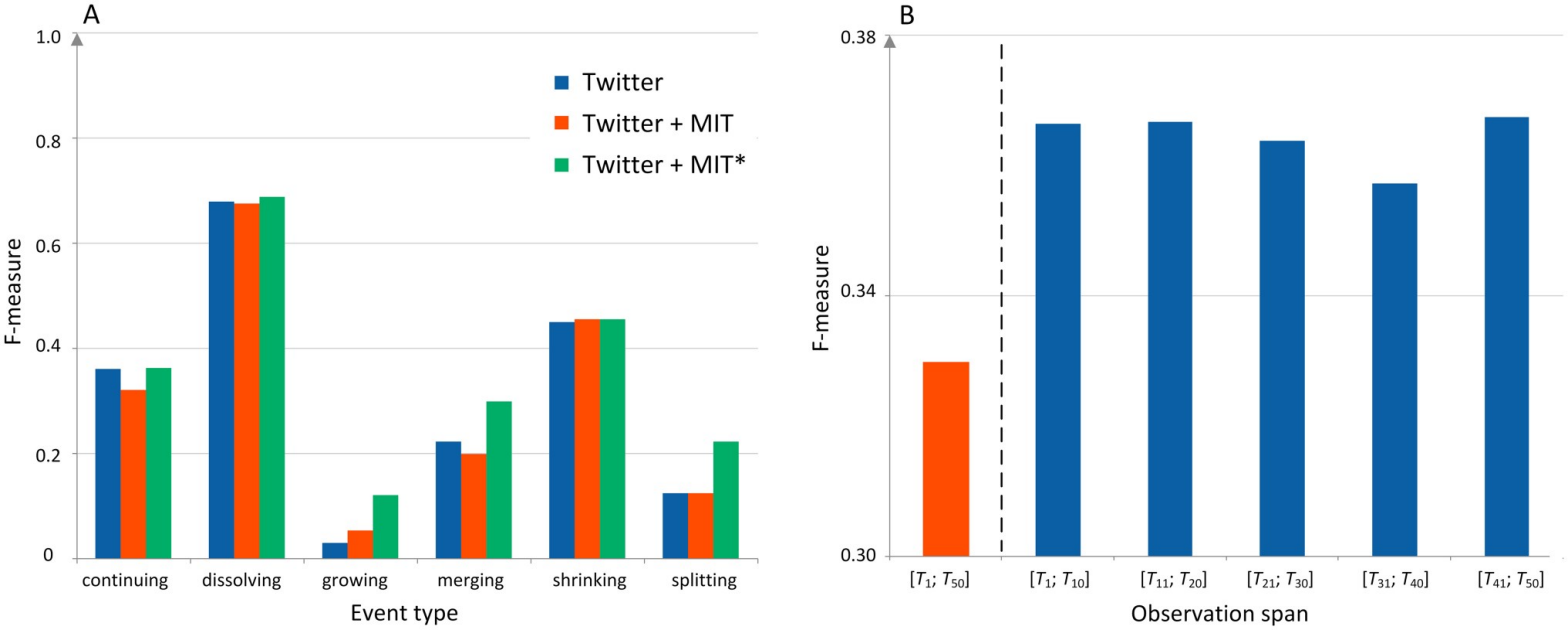
Application of the GEP method. **(A) Enriching the classification model** by partially balancing the original training set (Twitter) with extra evolution chains taken from another full external data set (MIT) or with chains from only selected event types, i.e. growing, merging and splitting (MIT*); chains with these classes were the worst classified events for the original Twitter data—they had the lowest F-measure values. The results for these selectively enriched event types were significantly improved without worsening classification for other classes (green vs. blue bars). Data enriching was performed only for learning, not for testing. **(B) Concept drift**. Classification quality for the Facebook data from one longer period *T*_1_ − *T*_50_ (the red bar); alternatively, the data was split into five smaller periods and separate classification models were built to catch concept drift phenomena between periods (blue bars). Independent models learned for smaller periods are better adapted to the changing environments.

Very promising results, although at an early stage, were achieved at enriching the learning phase of the classification model with additional evolution chains from a different data set. By partially balancing the original training set with extra evolution chains from another external data set, it was possible to improve the model and thus produce better results for minority classes, without affecting the outcome for the dominating classes, Fig 5A. This phenomenon is especially important because the existing techniques of balancing a data set always affect the classification results of the dominating classes.

Another way to enhance the classification model, initially considered, is an appropriate selection of the observation time span to reduce the effect of non-stationarity of data—a.k.a concept drift. Our preliminary research shows that for a network spanning over a long period or changing rapidly, updating the classification model every once in a while might improve the results, as the model reflects the current characteristics of the network in the better and more up to date way, Fig 5B. Nevertheless, in order to rebuild the model every now and then, the number of observations (evolution chains) extracted from such shorter time span must be high enough.

The GEP framework can be applied to any dynamic network data, i.e., to any complex network changing over time. In this paper, we have explored popular social network data, see Table B in S1 File. However, the entire GEP method, its stages and component solutions may be used for diverse complex networks [40, 41] like evolving clusters of web pages [42], co-citation and bibliographic coupling networks extracted from citations between scientific papers [43, 44], biological and medical networks [45, 46], linguistic networks linking word meanings—WordNets [47], multimedia networks [48] and many more.

## Conclusion

The main subject studied in this paper is group evolution prediction in social/complex networks. Its primary goal is to foresee a change like shrinking, growing, splitting, merging, or dissolving that the recently existing community will experience in the nearest future. To be able to perform any prediction, the most common approach is to process a temporal complex network *TSN* extracted from the stream of user activity traces. Communities and their changes are identified and predicted within such *TSN*. However, the existing methods are often limited to operate on a particular data set or to solve a specific problem, which makes them useful only in a particular and narrow domain.

Therefore, a new generic method called Group Evolution Prediction (GEP) has been proposed in this paper. The GEP method has a modular structure, which makes it very flexible and allows us to successfully apply it to any data set and under any specific requirements. The method consists of several stages; each of them involves a suitable selection of methods, algorithms, and attributes—the GEP method parameters. The evaluation process of the GEP method included: (1) analysis of numerous parameters (time window type and size, community detection method, evolution chain length, classifier used, set of features, and more), (2) comparative analysis against other existing methods, (3) adaptation of the transfer learning concept to group evolution prediction, (4) enriching the classification model with evolution chains from a different data set, and (5) enhancing the classification model with a more appropriate training set.

Regarding the time window types and sizes, the main finding is that for rapidly changing or sparse social networks a shorter overlapping time windows (in relation to the context of the data) are a better choice than longer or disjoint periods. On the contrary, if relations between individuals are recurrent and the network is rather dense, one may try disjoint time windows to obtain more concise results and to lower the computational cost. If long-lasting, persistent communities are the goal, then the increasing type of time window is the best choice as it generates a high number of the continuing, growing, and shrinking events.

Two most commonly used community detection approaches were analyzed: the CPM method detecting the overlapping communities, and the Infomap method identifying the disjoint communities. It turned out that the CPM method was not a proper choice for sparse networks, as it left out nodes that did not belong to any clique. However, if a network is not so sparse, then generating overlapping communities may be a better choice, especially if the context of the data suggests overlapping communities. For example, when the nodes tend to belong to more than one community at a given time. The Infomap method, however, performs better if computational complexity is an essential factor, and computational time is limited.

The results yield that evolution chains with more community states (longer chains) provide better classification results. However, there seems to be a threshold of the number of states, which make the evolution chains too short, resulting in a lack of possibility of improving the accuracy level.

Even over 70% of the most prominent features were obtained from the last three community states. It means that the most recent history of the community has the highest impact on its next change. This is an extremely useful conclusion if one has limited computational capabilities and cannot calculate community profiles for all states. Additionally, many new predictive features are proposed in this paper. In particular, some aggregations of node measures were used to compute the local and global microscopic features. Network structural measures were adopted as macroscopic features, and ratios of community measures to network measures were utilized as mesoscopic features. In general, the variations of the eigenvector-, eccentricity-, and closeness-based features were present in most of the selective rankings, which suggests that centrality- and distance-based measures obtained on the node level are the most valuable features.

The GEP method flexibility enabled us to investigate some other interesting scenarios, i.e., (1) adapting the transfer learning technique to the group evolution prediction problem, (2) enriching the classification model with evolution chains from a different data set, (3) appropriate selection of the observation time span to reduce the concept drift effect. All of them appeared to be quite successful.

Even though the GEP method is a flexible, generic framework, it is competitive with other approaches often dedicated to a specific problem or data set.

## Supporting information

S1 File Supporting information file. Contains additional results and discussion.
(PDF)

## References

[1] Zickenrott S, Angarica V, Upadhyaya B, Del Sol A. Prediction of disease–gene–drug relationships following a differential network analysis. Cell death & disease. 2017;7(1):e2040 10.1038/cddis.2015.393 PMC4816176 26775695

[2] Barabási AL, Gulbahce N, Loscalzo J. Network medicine: a network-based approach to human disease. Nature reviews genetics. 2011;12(1):56 10.1038/nrg2918 21164525 PMC3140052

[3] Wu X, Jiang R, Zhang MQ, Li S. Network-based global inference of human disease genes. Molecular systems biology. 2008;4(1):189 10.1038/msb.2008.27 18463613 PMC2424293

[4] Goh KI, Cusick ME, Valle D, Childs B, Vidal M, Barabási AL. The human disease network. Proceedings of the National Academy of Sciences. 2007;104(21):8685–8690. 10.1073/pnas.0701361104 PMC1885563 17502601

[5] Pauly MD, Procario MC, Lauring AS. A novel twelve class fluctuation test reveals higher than expected mutation rates for influenza A viruses. eLife. 2017;6 10.7554/eLife.26437 28598328 PMC5511008

[6] Parvin JD, Moscona A, Pan W, Leider J, Palese P. Measurement of the mutation rates of animal viruses: influenza A virus and poliovirus type 1. Journal of virology. 1986;59(2):377–383. 3016304 10.1128/jvi.59.2.377-383.1986 PMC253087

[7] Layne SP, Monto AS, Taubenberger JK. Pandemic influenza: an inconvenient mutation. Science. 2009;323(5921):1560–1561. 10.1126/science.323.5921.1560 19299601 PMC2778479

[8] Husnain M, Toor A. The Impact of Social Network Marketing on Consumer Purchase Intention in Pakistan: Consumer Engagement as a Mediator. Asian Journal of Business and Accounting. 2017;10(1):167–199.

[9] Antoniadis I, Charmantzi A. Social network analysis and social capital in marketing: theory and practical implementation. International journal of technology marketing. 2016;11(3):344–359. 10.1504/IJTMKT.2016.077387

[10] Guo L, Zhang M, Wang Y. Effects of customers’ psychological characteristics on their engagement behavior in company social networks. Social Behavior and Personality: an international journal. 2016;44(10):1661–1670.

[11] Barhemmati N, Ahmad A. Effects of Social Network Marketing (SNM) on Consumer Purchase Behavior throughCustomer Engagement. Journal of Advanced Management Science Vol. 2015;3(4).

[12] Kozinets RV, de Valck K, Wojnicki AC, Wilner SJS. Networked Narratives: Understanding Word-of-Mouth Marketing in Online Communities. Journal of Marketing. 2010;74(2):71–89.

[13] Palla G, Barabási AL, Vicsek T. Quantifying social group evolution. Nature. 2007;446(7136):664 10.1038/nature05670 17410175

[14] Bródka P, Saganowski S, Kazienko P. Tracking group evolution in social networks. In: International Conference on Social Informatics. Springer; 2011. p. 316–319.

[15] Saganowski S, Bródka P, Kazienko P. Community Evolution. Encyclopedia of Social Network Analysis and Mining. 2017; p. 1–14. 10.1007/978-1-4614-7163-9_223-1

[16] Rossetti G, Cazabet R. Community discovery in dynamic networks: a survey. ACM Computing Surveys (CSUR). 2018;51(2):35 10.1145/3172867

[17] Goldberg MK, Magdon-Ismail M, Nambirajan S, Thompson J. Tracking and Predicting Evolution of Social Communities In: SocialCom/PASSAT. Citeseer; 2011 p. 780–783.

[18] Qin G, Yang J, Gao L, Li J. Evolution pattern discovery in dynamic networks. In: Signal Processing, Communications and Computing (ICSPCC), 2011 IEEE International Conference on. IEEE; 2011. p. 1–6.

[19] Kairam SR, Wang DJ, Leskovec J. The life and death of online groups: Predicting group growth and longevity. In: Proceedings of the fifth ACM international conference on Web search and data mining. ACM; 2012. p. 673–682.

[20] Si C, Jiao L, Wu J, Zhao J. A group evolving-based framework with perturbations for link prediction. Physica A: Statistical Mechanics and its Applications. 2017;475:117–128. 10.1016/j.physa.2017.01.087

[21] Richter Y, Yom-Tov E, Slonim N. Predicting customer churn in mobile networks through analysis of social groups. In: Proceedings of the 2010 SIAM international conference on data mining. SIAM; 2010. p. 732–741.

[22] Xiao G, Zheng Z, Wang H. Evolution of Linux operating system network. Physica A: Statistical Mechanics and its Applications. 2017;466:249–258. 10.1016/j.physa.2016.09.021

[23] Atzmueller M, Ernst A, Krebs F, Scholz C, Stumme G. Formation and temporal evolution of social groups during coffee breaks In: Big Data Analytics in the Social and Ubiquitous Context. Springer; 2014 p. 90–108.

[24] Bródka P, Kazienko P, Kołoszczyk B. Predicting group evolution in the social network. Social Informatics. 2012; p. 54–67.

[25] Bródka P, Saganowski S, Kazienko P. GED: the method for group evolution discovery in social networks. Social Network Analysis and Mining. 2013;3(1):1–14. 10.1007/s13278-012-0058-8

[26] Gliwa B, Saganowski S, Zygmunt A, Bródka P, Kazienko P, Kozak J. Identification of group changes in blogosphere. In: Proceedings of the 2012 International Conference on Advances in Social Networks Analysis and Mining (ASONAM 2012). IEEE Computer Society; 2012. p. 1201–1206.

[27] Gliwa B, Bródka P, Zygmunt A, Saganowski S, Kazienko P, Koźlak J. Different Approaches to Community Evolution Prediction in Blogosphere. In: Proceedings of the 2013 IEEE/ACM International Conference on Advances in Social Networks Analysis and Mining. ASONAM’13. New York, NY, USA: ACM; 2013. p. 1291–1298. Available from: http://doi.acm.org/10.1145/2492517.2500231.

[28] Ilhan N, Oguducu IG. Community event prediction in dynamic social networks. In: Machine Learning and Applications (ICMLA), 2013 12th International Conference on. vol. 1. IEEE; 2013. p. 191–196.

[29] Takaffoli M, Rabbany R, Zaïane OR. Community evolution prediction in dynamic social networks. In: Advances in Social Networks Analysis and Mining (ASONAM), 2014 IEEE/ACM International Conference on. IEEE; 2014. p. 9–16.

[30] Saganowski S, Gliwa B, Bródka P, Zygmunt A, Kazienko P, Koźlak J. Predicting community evolution in social networks. Entropy. 2015;17(5):3053–3096. 10.3390/e17053053

[31] Diakidis G, Karna D, Fasarakis-Hilliard D, Vogiatzis D, Paliouras G. Predicting the evolution of communities in social networks. In: Proceedings of the 5th International Conference on Web Intelligence, Mining and Semantics. ACM; 2015. p. 1.

[32] İlhan N, Öğüdücü ŞG. Feature identification for predicting community evolution in dynamic social networks. Engineering Applications of Artificial Intelligence. 2016;55:202–218. 10.1016/j.engappai.2016.06.003

[33] Pavlopoulou MEG, Tzortzis G, Vogiatzis D, Paliouras G. Predicting the evolution of communities in social networks using structural and temporal features. In: Semantic and Social Media Adaptation and Personalization (SMAP), 2017 12th International Workshop on. IEEE; 2017. p. 40–45.

[34] Palla G, Derényi I, Farkas I, Vicsek T. Uncovering the overlapping community structure of complex networks in nature and society. Nature. 2005;435(7043):814–818. 10.1038/nature03607 15944704

[35] Rosvall M, Bergstrom CT. Maps of random walks on complex networks reveal community structure. Proceedings of the National Academy of Sciences. 2008;105(4):1118–1123. 10.1073/pnas.0706851105 PMC2234100 18216267

[36] He Z, Tajeuna EG, Wang S, Bouguessa M. A Comparative Study of Different Approaches for Tracking Communities in Evolving Social Networks. In: Data Science and Advanced Analytics (DSAA), 2017 IEEE International Conference on. IEEE; 2017. p. 89–98.

[37] Hopcroft J, Khan O, Kulis B, Selman B. Tracking evolving communities in large linked networks. Proceedings of the National Academy of Sciences. 2004;101(suppl 1):5249–5253. 10.1073/pnas.0307750100 PMC387303 14757820

[38] Friedman M. The use of ranks to avoid the assumption of normality implicit in the analysis of variance. Journal of the american statistical association. 1937;32(200):675–701. 10.1080/01621459.1937.10503522

[39] Shaffer JP. Modified sequentially rejective multiple test procedures. Journal of the American Statistical Association. 1986;81(395):826–831. 10.1080/01621459.1986.10478341

[40] Barzel B, Barabási AL. Universality in network dynamics. Nature physics. 2013;9:673–681. 10.1038/nphys2741 PMC3852675 24319492

[41] Boccaletti S, Latora V, Moreno Y, Chavez M, Hwang DU. Complex networks: Structure and dynamics. Physics Reports. 2006;424(4):175–308. 10.1016/j.physrep.2005.10.009.

[42] Dezsö Z, Almaas E, Lukács A, Rácz B, Szakadát I, Barabási AL. Dynamics of information access on the web. Phys Rev E. 2006;73:066132 10.1103/PhysRevE.73.066132 16906939

[43] Kessler MM. Bibliographic coupling between scientific papers. American Documentation. 1963;14(1):10–25. 10.1002/asi.5090140103

[44] Small H. Co-citation in the scientific literature: A new measure of the relationship between two documents. Journal of the American Society for Information Science. 1973;24(4):265–269. 10.1002/asi.4630240406

[45] Böde C, Kovács IA, Szalay MS, Palotai R, Korcsmáros T, Csermely P. Network analysis of protein dynamics. FEBS Letters. 2007;581(15):2776–2782. 10.1016/j.febslet.2007.05.021 17531981

[46] Barabási AL, Gulbahce N, Loscalzo J. Network medicine: a network-based approach to human disease. Nature reviews Genetics. 2011;12(1):56–68. 10.1038/nrg2918 21164525 PMC3140052

[47] Bartusiak R, Łukasz Augustyniak, Kajdanowicz T, Kazienko P, Piasecki M. WordNet2Vec: Corpora agnostic word vectorization method. Neurocomputing. 2019;326-327:141–150. 10.1016/j.neucom.2017.01.121.

[48] Indyk W, Kajdanowicz T, Kazienko P. Relational large scale multi-label classification method for video categorization. Multimedia Tools and Applications. 2013;65(1):63–74. 10.1007/s11042-012-1149-2

