# Supplementary material for: Analysis of group evolution prediction in complex networks
Source: PLoS One. 2019 Oct 29;14(10):e0224194. doi: 10.1371/journal.pone.0224194 (PMC6818769; doi:10.1371/journal.pone.0224194)
Supplement: S1 File — Contains additional results and discussion. (PDF) [file pone.0224194.s001.pdf]

Analysis of group evolution prediction in complex networks - supplementary information

Stanisław Saganowski[1,*], Piotr Bródka[1], Michał Koziarski[2], Przemysław Kazienko[1]

1 Department of Computational Intelligence, Faculty of Computer Science and Management, Wrocław University of Science and Technology, Wrocław, Poland
2 Department of Electronics, Faculty of Computer Science, Electronics and Telecommunications, AGH University of Science and Technology, Kraków, Poland

* stanislaw.saganowski@pwr.edu.pl

Example of a social group on Facebook

The Facebook platform allows to perform various social activities like discussion in groups, content sharing, commenting, expressing opinions and emotions. One of the platform's tools allows to create and join independent discussion groups devoted to a specific topic. For example, there are groups intended for mothers living in Singapore, which purpose is to talk about and comment on new products for babies, share general advices about raising children, sell used clothes, etc.

By obtaining and processing data of a single discussion group we are able to create its social network graph, and furthermore, we can track its evolution. Depending on what we are trying to achieve, we would process data in a different way. In the simplest case we can assume that one post (content posted to the discussion group) and all interactions to this post (likes, comments, shares) reflect a social group at a particular time. By obtaining social groups for each post we can create a temporal social network of the considered discussion group and analyze its activeness over time. In a more complex scenario, we can analyze the content of the comments and types of interactions within each post to discover two or more groups with different opinions, e.g. recommending and criticizing a new product for babies.

Group Evolution Prediction methods

The summary of the most relevant methods for group evolution prediction known from the literature confronted with GEP, which is described in this paper, can be found in Tab. A.

Data sets used

Fifteen real-world data sets were analyzed in the iterative process of evaluating and improving the GEP method. Nonetheless, the results presented in this work refer to ten out of fifteen analyzed data sets. The limitation was made to keep the paper clear and concise. The data sets were selected in such way, that the networks created from them had diverse characteristics, see Tab. B. During the experimental studies, the parameters of the GEP method and its components (algorithms, methods, tools) were adjusted based on the literature review, authors suggestion, previous results and experience, and sometimes as a result of repeating the experimental study endless number of times.

Table A. Methods for group evolution prediction.

Method name	Time window type	Type of communities	Community evolution tracking method	No. of predictive features per group state	No. of classifiers analyzed	No. of real-world data sets analyzed	Prediction goal
GEP	any	any	any	91	15	15	next event (6 classes), several forthcoming events, community measure
İlhan et al. [1, 2]	increasing	disjoint	included in the method	9	10	4 + 40 synthetic	next event (6 classes)
Takaffoli et al. [3]	disjoint	disjoint	MODEC	33	9	2	next event (3 classes), size, cohesion
Diakidis et al. [4]	overlapping	overlapping	GED	10	7	1	next event (4 classes)
Goldberg et al. [5, 6]	disjoint	overlapping	included in the method	20 (average)	1	2	length of community lifetime
Kairam et al. [7]	disjoint	unknown	unknown	8	1	1	community growth rate and longevity (binary classification)

Table B. Characteristics of the data sets used in the research

Data set name	Source	Nodes	Edges	Avg. degree	Time span	Directed	Short description
DBLP	[8]	1,314,050	18,986,618	28.9	20 years	no	co-authorship of articles from the DBLP bibliography
Digg	[9]	30,398	87,627	5.8	15 days	yes	replies between users on the Digg online platform
Facebook	[10]	46,952	876,993	37.4	1 year	yes	posts to other user's wall on the Facebook social platform
Infectious	[11]	410	17,298	84.4	8 hours	no	face-to-face contacts during an exhibition
IrvineForum	[12, 13]	899	33,700	74.0	164 days	no	students activity on the UC Irvine discussion forum
IrvineMessages	[12]	1,899	59,835	63.0	6 months	yes	private messages between students of the UC Irvine
Loans	[14]	89,269	3,394,979	76.1	1 year	yes	loans between users of the prosper.com platform
MIT	[15]	96	1,086,404	22.6	9 months	no	face-to-face contacts between students of the MIT
Slashdot	[16]	51,083	140,778	5.5	32 months	yes	replies between users on the Slashdot online portal
Twitter	[13, 17]	18,500	61,200	6.0	48 days	no	retweets between users on the Twitter social platform

Evolution chain duplication

Let's consider creating evolution chains of length 2 for the exemplary community evolution depicted in Fig. A. The list of evolution chains would contain five unique pairs

of following states, see Tab. C. As one can observe, some evolution chains are partially duplicated, e.g., state ST_2 and event EV_2 of chain EC_1 are the same as state ST_1 and event EV_1 of chain EC_2, chains EC_2 and EC_3 have the same state ST_1 and event EV_1, chains EC_4 and EC_5 share the same state ST_2 and event EV_2, and so on. The number of duplicated states and events would be even higher for a longer evolution chains. The partial duplication is a result of mixing (crossing) lifetimes of several groups, as the splitting and merging events involve at least two communities from the same time window: $G_{1,3}$ and $G_{2,3}$ in this example.

Even partially duplicated chains might be a problem, as they may affect the classification results. For example, if chains EC_2 and EC_4 would be in the training set, used to learn a classifier, and chains EC_3 and EC_5 would be in the test set, used to evaluate the classification model, the classification accuracy for chains EC_3 and EC_5 could be falsely improved, because the classifier might assign the correct event type based on "remembering" the data, rather than learning from them. One may try to remove the partially duplicated chains by applying procedure similar to the "group by" SQL command. However, this will always result in losing some information as well. In this example, grouping chains on state ST_2 and event EV_2 would result in removing chain EC_5.

A better solution is to use single-state chains, see Tab. D for the set of chains obtained from the considered exemplary evolution. Single-state chains can also contain duplicated states, e.g., when a method for tracking evolution will assign two different event types to the same community, but it is a rare case, and such duplication can be easily removed. Throughout this paper, the process of removing partially duplicated chains is called "removing duplicates."

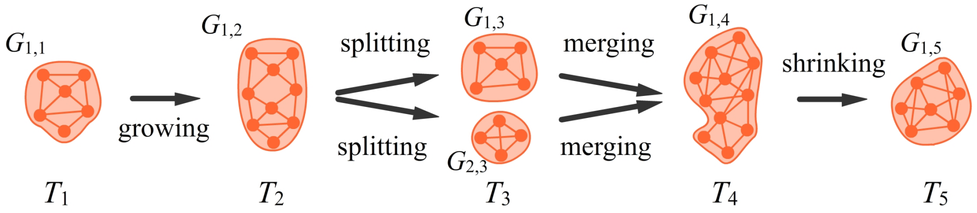

Fig A. An example of community evolution containing five states and four events.

Table C. Evolution chains of length 2 created from the community evolution presented in Fig. A.

Evolution chain	Group state ST_1 in T_i	Event type EV_1	Group state ST_2 in T_{i+1}	Event type EV_2
EC_1	$G_{1,1}$	growing	$G_{1,2}$	splitting
EC_2	$G_{1,2}$	splitting	$G_{1,3}$	merging
EC_3	$G_{1,2}$	splitting	$G_{2,3}$	merging
EC_4	$G_{1,3}$	merging	$G_{1,4}$	shrinking
EC_5	$G_{2,3}$	merging	$G_{1,4}$	shrinking

Feature selection

Feature extraction is an essential step that needs to be performed prior to the classification. Various measures can be used to represent the characteristic of the community at any given time step. Much effort has been made by various researchers to propose such measures, leading to their abundance. However, the high number of features is not always beneficial in the classification process. It can lead to the necessity

Table D. Single-state evolution chains created from the community evolution presented in Fig. A.

Evolution chain	Group state ST_1 in T_i	Event type EV_1
EC_1	$G_{1,1}$	growing
EC_2	$G_{1,2}$	splitting
EC_3	$G_{1,3}$	merging
EC_4	$G_{2,3}$	merging
EC_5	$G_{1,4}$	shrinking

of obtaining more data for training, which is not always feasible. Not all classifiers are resilient to the presence of uninformative features, which can weaken their performance. Finally, feature extraction can be a time consuming procedure, during both training and evaluation of the model. Due to abovementioned factors, the number of utilized features should ideally be kept to the minimum, as long as it does not lead to loss in performance.

To address this issue, feature selection process [18] can be performed prior to classification. Feature selection is a procedure of automatically selecting a subset of features from the larger set, possibly containing irrelevant or mutually redundant features. The aim of such task is twofold: to improve the performance of the trained model, as well as to reduce the evaluation time during its testing. However, feature selection does not address the issue of long training time. On the contrary, based on the chosen method of selection, training can be significantly prolonged. Furthermore, feature selection might itself require large amounts of data to lead to meaningful results, instead of overfitting to the task at hand. Finally, feature selection by itself gives little insight into the problem. Selected features may or may not generalize well to the other, related problems, which in uncertain when the selection is performed on a single dataset.

In the experiment described in this section, we implemented a slightly different task – feature ranking, with the aim of providing more insight about all considered measures. Given a large number of benchmark datasets, we tried to evaluate which measures lead to the best performance during the classification. To this end, we performed a feature selection based on the evolutionary algorithm [19]. This procedure was repeated for various datasets and random data partitionings. Finally, we constructed a feature rankings based on the frequency of the occurrence of the given feature in the final selection. Because the feature selection strategy aims to optimize the classification performance, we postulate that the produced rankings indicate the quality of the features in the group evolution prediction task, with the quality being defined as an expected performance on the problems from the same domain. To the best of our knowledge, such evaluation has not been done before in the context of social group prediction. In the remainder of this section, we describe the proposed method more in-depth along with the most significant results.

Method

The goal of the feature selection procedure is selecting a subset of features maximizing classification performance, at the same time minimizing the cost (most often computational) of producing the final subset. Given specific performance and cost measures, as well as the weights associated with both of these factors, in principle, it is possible to find the optimal feature subset, at least with regard to the available data. However, individually valuable features, i.e. the ones leading to the highest performance if used as the only predictor, will not necessarily be a part of the optimal subset. It has been shown [18] that the feature useless by itself can improve performance significantly when taken with others, and that the presence of highly correlated features can negatively affect the performance. Therefore, if finding the optimal feature subspace

was possible, a distinction between high-quality features (those included in the optimal subset) and low-quality features (the remaining ones) can be made. However, as the number of the available features grows larger, evaluating all of the possible subsets becomes infeasible. Instead of the optimal feature subset, one has to rely on its approximation produced by the feature selection procedure. If numerous such approximations can be produced, one can associate feature quality with the frequency of the occurrence of the feature in the selected subset. Similarly, the optimality can be discussed only with regard to the available data, which is only an approximation of the underlying distribution. By selecting different data sample, we obtain a different feature subset, which is only an approximation of the optimal one.

We propose associating individual feature quality with the fraction of time it appears in the selected feature subset. On the data level, we provide diversity in the produced feature subsets by performing 5×2-fold partitioning [20] on the original dataset. Furthermore, we perform a non-deterministic feature selection using basic evolutionary algorithm [21] and repeat it multiple times with a random initialization. The goal of the evolutionary algorithm is selecting a feature subset optimizing the defined fitness function.

Let us denote the original data by a tuple (X, y), with X being a $n \times d$ dimensional matrix of n observations consisting of d features each, and y being a vector of n class labels associated with observations. Furthermore, let us denote a d-dimensional binary mask encoding which features are present in the selected subset by $\hat{s}$, with $\hat{s}_i$ indicating the presence of the ith feature. Finally, let us denote by $X^{(\hat{s})}$ the subselection of the observations, consisting only of the features encoded in $\hat{s}$. Given the partitioning of (X, y) into the training data (X_{train}, y_{train}), validation data (X_{val}, y_{val}) and test data (X_{test}, y_{test}), we denote the weighted F_1 score obtained by training the classifier on subselection $(X^{(\hat{s})}_{train}, y_{train})$ and evaluating its performance on subselection $(X^{(\hat{s})}_{val}, y_{val})$ as $F_1(\hat{s})$. We can then define the final fitness function, optimized by the evolutionary algorithm, as

$$f(\hat{s}) = \gamma \times F_1(\hat{s}) - \delta \times \frac{\sum_{i=1}^{d} \hat{s}_i}{d}, \tag{1}$$

with γ being the coefficient assigned to the classification performance, and δ – the coefficient assigned to the number of the selected features. The evolutionary algorithm using such fitness function performs a multi-objective optimization, with the objectives: maximize the classification performance and minimize the number of selected features, and the weight associated to the objectives based on the choice of γ and δ.

For the experiments, the values of γ and δ have been set to 0.8 and 0.2, respectively. They were chosen to keep the number of features in a given selection relatively small, with the exact value dependent on the dataset. The Random Forest was chosen as the classifier used to evaluate the classification performance of a given feature subset. The original data has been split into the training, validation and test partitions in the proportion of 0.375, 0.125 and 0.5, respectively. Finally, the following parameters of the evolutionary algorithm have been used: number of generations of 100, population size of 500, probability of mutation of 0.02, probability of crossover of 0.7, and the tournament selection with the size of 3. For each of the 5×2 folds, the evolutionary algorithm has been run 100 times, leading to 1000 feature subsets, based on which the final feature rankings have been computed.

During the conducted experimental study, all GEP features (Tab. L), and additionally the features proposed by İlhan et al. in [2] were analyzed. The features were obtained from 7 real-world data sets: Digg, Facebook, Infectious, IrvineMessages, Loans, MIT, Slashdot, see Tab. B. The Infomap method was applied to obtain the disjoint communities, which evolution was then tracked by means of the GED method with the alpha and beta parameters set to 50%. Time windows of various type and size,

as well as the evolution chains of various length, were used to evaluate abovementioned data sets, which led to 28 separate rankings. See Tab. E for the detailed information about the data setup parameters. For each configuration from Tab. E, a separate ranking was created. However, to draw more general conclusions some rankings were merged together by averaging occurrences of features in separate rankings. Only rankings containing the same set of features can be merged, thus, the same length of the evolution chain is required. Therefore, the merged rankings were obtained from the evolution chains of the following lengths: all 1-state evolution chains - Tab. F (ids 1-12 in Tab. E), all 2-state evolution chains - Tab. G (ids 13-18 in Tab. E), all 3-state evolution chains - Tab. H (ids 19-24 in Tab. E), and all 9-state evolution chains - Tab. I (ids 26, 27, 28 in Tab. E).

Table E. The configuration of parameters utilized to obtain 28 feature quality rankings

Ranking id	Evolution chain length	Time window type	Data set	Time window size	No. of time windows
1	1 state	disjoint	Infectious	s=15min	32
2		overlapping	Facebook	s=28days; o=14days	27
3		overlapping	IrvineMessages	s=2days; o=1day	192
4		overlapping	IrvineMessages	s=7days; o=3days	47
5		overlapping	IrvineMessages	s=14days; o=7days	26
6		overlapping	IrvineMessages	s=28days; o=14days	12
7		overlapping	Loans	s=30days; o=15days	23
8		overlapping	MIT	s=7days; o=3days	57
9		overlapping	MIT	s=30days; o=15days	14
10		increasing	Digg	s=2days	10
11		increasing	MIT	s=30days	10
12		increasing	Slashdot	s=36days	10
13	2 states	disjoint	Infectious	s=15min	32
14		overlapping	IrvineMessages	s=7days; o=3days	47
15		overlapping	IrvineMessages	s=14days; o=7days	26
16		overlapping	IrvineMessages	s=28days; o=14days	12
17		overlapping	Loans	s=30days; o=15days	23
18		overlapping	MIT	s=30days; o=15days	14
19	3 states	overlapping	Facebook	s=28days; o=14days	27
20		overlapping	IrvineMessages	s=2days; o=1day	192
21		overlapping	MIT	s=7days; o=3days	57
22		increasing	Digg	s=2days	10
23		increasing	MIT	s=30days	10
24		increasing	Slashdot	s=36days	10
25	8 states	increasing	Digg	s=2days	10
26	9 states	overlapping	Facebook	s=28days; o=14days	27
27		overlapping	MIT	s=7days; o=3days	57
28		increasing	Slashdot	s=36days	10

In summary, the rankings of the most prominent features were different between various data sets and types of the time window, since the characteristics of the obtained temporal social networks were different. However, it was possible to identify a few measures, which appeared more often in the top ten features of various rankings. The variations of the eigenvector-, eccentricity-, and closeness-based measures were present in most of the presented shortlisted rankings, which suggests that centrality- and distance-based measures, obtained at the node level, are more informative predictors for the classifier. Surprisingly, measures describing the community in the most straightforward way, e.g., the community size or density, did not occur in the shortlisted

Table F. The top ten features of the merged rankings for all 1-state evolution chains (ids 1-12 in Tab. E). Bolded features are newly proposed.

Rank	Feature	Occurrences	Feature type
1	IlhanAging	258	microscopic
2	network_density	242	macroscopic
3	**network_leadership**	216	macroscopic
4	**avg_group_eccentricity**	185	microscopic local
5	**sum_network_closeness**	178	microscopic global
6	**min_group_eigenvector**	164	microscopic local
7	**max_network_degree_total**	162	microscopic global
8	**network_reciprocity**	146	macroscopic
9	**max_group_closeness**	146	microscopic local
10	**max_network_closeness**	145	microscopic global

Table G. The top ten features of the merged rankings for all 2-state evolution chains (ids 13-18 in Tab. E). Bolded features are newly proposed.

Rank	Feature	Occurrences	Feature type
1	**avg_group_eccentricity** T_{n-1}	312	microscopic local
2	**beta** T_{n-1}	293	mesoscopic
3	**alpha** T_{n-1}	261	mesoscopic
4	group_coefficient_global T_{n-1}	259	mesoscopic
5	**network_ratio_coefficient_global** T_{n-1}	258	mesoscopic
6	**sum_group_closeness** T_{n-1}	251	microscopic local
7	**sum_group_betweenness** T_{n-1}	243	microscopic local
8	network_density T_{n-1}	242	macroscopic
9	**avg_group_closeness** T_{n-1}	222	microscopic local
10	**max_group_closeness** T_{n-1}	218	microscopic local

Table H. The top ten features of the merged rankings for all 3-state evolution chains (ids 19-24 in Tab. E). Bolded features are newly proposed.

Rank	Feature	Occurrences	Feature type
1	**beta** T_{n-1}	590	mesoscopic
2	**avg_group_eccentricity** T_{n-1}	403	microscopic local
3	**min_group_eigenvector** T_{n-1}	374	microscopic local
4	**beta** T_{n-2}	344	mesoscopic
5	**network_ratio_eccentricity** T_{n-1}	342	mesoscopic
6	**avg_network_degree_total** T_{n-1}	322	microscopic global
7	**avg_group_eigenvector** T_{n-1}	307	microscopic local
8	IlhanInter T_{n-1}	300	microscopic local
9	**avg_network_degree_in** T_{n-1}	290	microscopic global
10	**max_group_closeness** T_{n-1}	287	microscopic local

rankings, usually taking place in the second half of the rankings. Furthermore, the macroscopic features, especially the network density, were important only when the history of the community was very short (1-2 states). Thus, when there were more historical data available, classifiers preferred past microscopic and mesoscopic features over the recent macroscopic features. What is more, the predictive features proposed by İlhan et al. in [2] were ranked rather low, except the IlhanAging feature, which was the most commonly used in case of the 1-state evolution chains (Tab. F) and was usually also among the top 30 features in other rankings.

Table I. The top ten features of the merged rankings for all 9-state evolution chains (ids 26, 27, 28 in Tab. E). Bolded features are newly proposed.

Rank	Feature	Occurrences	Feature type
1	**min_group_eigenvector** T_{n-1}	521	microscopic local
2	**network_ratio_eccentricity** T_{n-1}	433	mesoscopic
3	**sum_network_eigenvector** T_{n-1}	428	microscopic global
4	**avg_network_eigenvector** T_{n-1}	427	microscopic global
5	**neighborhood_out** T_{n-1}	411	mesoscopic
6	**avg_group_eccentricity** T_{n-1}	403	microscopic local
7	**sum_network_betweenness** T_{n-1}	399	microscopic global
8	**neighborhood_all** T_{n-1}	398	mesoscopic
9	**neighborhood_in** T_{n-1}	395	mesoscopic
10	**avg_network_degree_in** T_{n-1}	395	microscopic global

Reproducibility

Experiment described in this section has been implemented in the Python programming language. Existing implementations of the classification algorithms from scikit-learn [22] and evolutionary algorithms from DEAP [23] have been used. Code sufficient to repeat the experiment has been made publicly available at[1], whereas the necessary data, especially its partitioning used during the experiment, has been provided at [24].

Classifiers used in the experiments

In this experimental study 15 different classifiers, implemented in WEKA Data Mining Software [25], were compared in term of the average F-measure value. They were gathered into six more general types.

Rule classifiers

- **ZeroR** is the simplest classification method, which relies on the target and ignores all predictors. ZeroR classifier simply classifies the majority category (class). Although there is no predictability power in ZeroR, it is useful for determining a baseline performance as a benchmark for other classification methods.

- **RIPPER** (JRip) is a propositional rule learner, also called Repeated Incremental Pruning to Produce Error Reduction (RIPPER), which was proposed by Cohen [26].

- **DecisionTable** builds a simple decision table majority classifier [27]. It evaluates the feature subsets using a best-first search and can use a cross-validation for the evaluation.

Function classifier

- **Support Vector Machine** (SVM) performs classification by finding the hyperplane that maximizes the margin between classes. The vectors (cases) that define the hyperplane are the support vectors [28].

[1] `https://github.com/michalkoziarski/SocialNetworkFeatureRanking`

Tree classifiers

- **REPTree** is a fast decision tree learner, which builds a decision/regression tree using the information gain/variance and prunes it using a reduced-error pruning (with backfitting). It only sorts values for the numeric attributes once, and the missing values are dealt with by splitting the corresponding instances into pieces.
- **RandomForest** is a well-known classifier for constructing a forest of random trees [29].
- **C4.5** (J48) is a classic classifier generating a pruned or unpruned C4.5 decision tree [30].
- **SimpleCart** is a classifier implementing the minimal cost-complexity pruning [31].

Bayes classifiers

- **NaiveBayes** is a simple classifier using estimator classes; numeric estimator precision values are chosen based on analysis of the training data [32].
- **BayesNet** is a factored representation of the probability distributions that generalize the naive Bayesian classifier and explicitly represent statements about independence [25].

Lazy classifiers

- **KNN** (IBk) is a simple algorithm that stores all available cases and classifies new cases based on a similarity measure, e.g., distance functions [33].
- **K*** KStar is an instance-based classifier, that is the class of a test instance is based upon the class of those training instances similar to it, as determined by some similarity function. It differs from other instance-based learners in that it uses an entropy-based distance function [34].

Meta-classifiers

- **AdaBoost** (with DecisionStump) is a classifier for boosting a nominal class classifier using the Adaboost M1 method [35]. DecisionStump [36] performs the classification based on entropy; missing values are treated as a separate value.
- **Bagging** (with REPTree) bags a classifier to reduce the variance. Can do classification and regression depending on the base learner [37].
- **MultiClassClassifier** (with Logistic) is a meta-classifier for handling multi-class data sets with 2-class classifiers. This classifier is also capable of applying error correcting output codes for increased accuracy. Logistic is a classifier building a multinomial logistic regression model with a ridge estimator [38].

Statistical tests of classifiers

In order to statistically compare classifiers the Friedman test [39] with the Shaffer post-hoc multiple comparisons [40] was utilized. The non-parametric statistical analysis was computed with the KEEL software tool [41]. The Friedman procedure was applied two times, once on the results obtained from the imbalanced data sets, and once on the results obtained from the data sets balanced with the equal size sampling technique.

Tab. J presents the average ranks obtained by applying the Friedman procedure. The test conducted on the imbalanced data sets produced p-value=$5.25 * 10^{-5}$, while the test on the balanced data sets provided p-value=$1.1 * 10^{-3}$. Since both p-values are much lower than 0.05, the results can be considered statistically significant.

In both cases, the Bagging classifier achieved the best ranks, and the RandomForest classifier was ranked second, while the ZeroR, AdaBoost and SVM classifiers performed worst. However, the Friedman test compares only the average F-measure values obtained for all event types, it does not take into account the fact that some of the events were not classified by the particular classifier, which may be crucial if a successful classification of all event types is the goal. For instance, the Bagging classifier, which achieved the highest ranks, was not able to classify: (1) the growing event for the imbalanced Twitter data set (Fig. BA), (2) the merging event for the balanced Twitter data set, and (3) the splitting event for the imbalanced Facebook data set. At the same time, the RandomForest classifier was able to classify all event types within data sets analyzed in this experiment (Fig. BB).

However, the post-hoc comparison revealed that the difference between the Bagging and RandomForest classifiers was not statistically significant. In fact, the difference between any tree classifier and the Bagging classifier was not statistically significant.

Table J. The average ranks of classifiers obtained by applying the Friedman procedure

Imbalanced data sets		Balanced data sets	
Algorithm	Avg. Ranking	Algorithm	Avg. Ranking
Bagging	1.00	Bagging	3.00
RandomForest	3.75	RandomForest	4.00
REPTree	3.75	BayesNet	5.50
C4.5	5.50	DecisionTable	5.75
MultiClassClassifier	5.50	REPTree	6.00
DecisionTable	6.75	SimpleCart	6.00
K*	6.75	NaiveBayes	6.75
SimpleCart	7.00	KNN	6.75
BayesNet	7.75	MultiClassClassifier	7.25
KNN	9.75	C4.5	7.75
RIPPER	10.50	K*	9.00
NaiveBayes	10.50	RIPPER	10.25
LibSVM	13.00	AdaBoost	13.25
AdaBoost	13.50	LibSVM	14.00
ZeroR	15.00	ZeroR	14.75

Adjusting classifiers parameters

A final step in the process of applying the machine learning is tuning the classifiers' parameters. This allows us to adjust the model to a given problem – data set, however, it also requires a lot of efforts to run thousands of iterations slightly modifying one parameter at a time. Therefore, only the top-ranked classifiers, i.e., the Bagging, RandomForest and C4.5 classifiers, were chosen for this experimental study and only one parameter per classifier was evaluated.

The result of tuning the Bagging classifier is presented in Fig. CA. The correlation between the number of bagging iterations and the average F-measure demonstrates a logarithmic tendency. The average F-measure value increased substantially from 0.305 with one bagging iteration to 0.360 with 50 bagging iterations. However, the additional bagging iterations are very costly in terms of computational time. It took over 24 hours

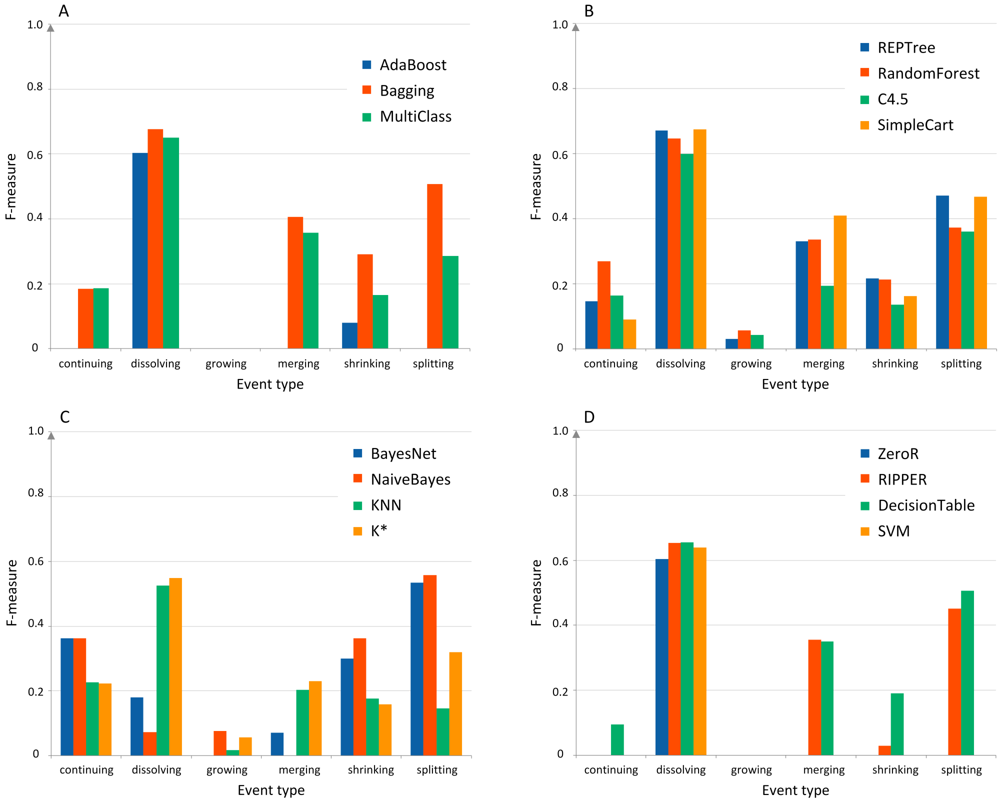

Fig B. The classification results of different classifiers for the 1-state evolution chains obtained from the imbalanced Twitter data set. **(A)** Meta-classifiers. **(B)** Tree classifiers. **(C)** Bayes and lazy classifiers. **(D)** Rule and function classifiers.

to obtain the classification results for the Bagging classifier with 50 bagging iterations and for this reason it was not further increased. Nonetheless, since the correlation between the number of bagging iterations and the average F-measure value has a logarithmic nature, it is enough to set the parameter to 10 (default value) or 20 in order to obtain a score close to the results obtained with 50 bagging iterations.

The number of generated trees by the RandomForest classifier also reveals the logarithmic correlation to the average F-measure value, Fig. CB. The overall classification score achieved with just one tree was 0.291, while the result obtained with 100 generated trees was as high as 0.350. Again, increasing the parameter value required a much longer computational time. Therefore, the experiment was discontinued for higher values. Based on the results, the parameter set to 50 seems to be a reasonable choice between the average F-measure value and the computational cost required to generate more trees. The overall score achieved with 50 trees was higher by 0.018 in comparison to the result obtained with the default parameter value (10 trees).

The confidence factor parameter of the C4.5 classifier was also correlated with the average F-measure value, see Fig. CC. Increasing the parameter value resulted in only a slight decrease of the overall score. The highest observed F-measure value was 0.326 and it was achieved with the confidence factor equal to 0.01, while the lowest overall score was 0.297, obtained with confidence of 0.99. The difference between the result achieved with the default parameter value (0.25) and the best result obtained with the parameter value set to 0.01 was 0.026.

In general, tuning the classifiers' parameters can yield notable differences in

F-measure values. Therefore, if the computational time is not limited, one may try to improve the classification results by adjusting classifiers' parameters. In combination with other improvements, the overall gain might be very significant.

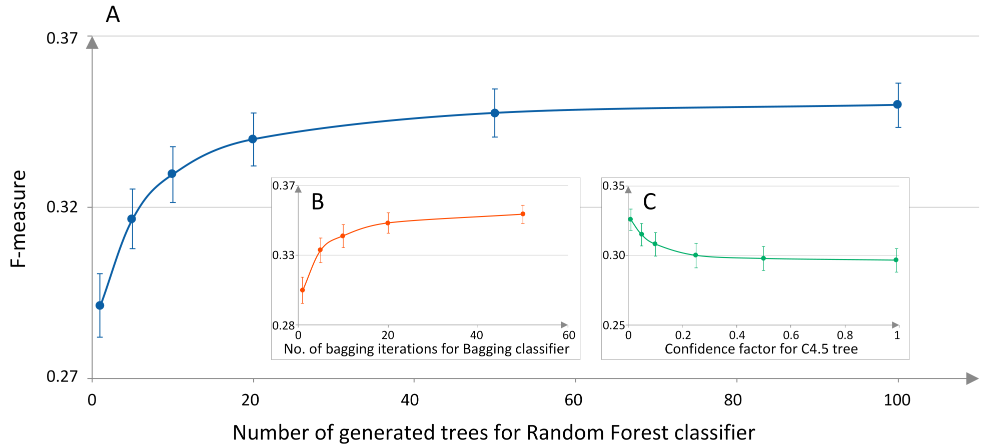

Fig C. The influence of classifiers' parameters adjustment on the F-measure value for the Facebook data set. **(A)** Tunning the number of generated trees in the Random Forest classifier. **(B)** Adjusting the number of bagging iterations in the Bagging classifier. **(C)** Fixing the confidence factor in the C4.5 tree.

Classification performance measure

Many measures capturing the classification performance have been proposed and evaluated [42–46]. The most often used measures for binary classification are: accuracy, precision, recall, fscore (F-measure), specificity, and AUC (Area Under the Curve); while for multi-class classification commonly are used: average accuracy, error rate, precision, recall, and fscore (F-measure) [44]. The formulas for all the measures are in Eq. 2-11.

In our study, the F-measure value (which is the harmonic mean of precision and recall) was utilized to indicate the classification performance for the particular class. Additionally, the average of all classes' F-measure values was computed to denote the overall classification quality. The reason for using the plain average F-measure instead of the weighted F-measure, globally averaged F-measure (macro- or micro-averaged [46]), or other measures as the overall score, was to emphasize the lack of classification of some classes better. Furthermore, the plain average F-measure value considers each class to be equally important. Hence, the results of the minority classes are not lost, like in case of the accuracy measure or weighted average F-measure. What is more, the total accuracy might be sometimes misleading, e.g., when one event type suppresses others. In such cases the classifier assigns the dominating type of the event to all observations to increase the accuracy, thus, resulting in a high number of true positive and true negative classifications.

See Tab. K for three samples of classification results and various overall performance measures computed for these samples. The plain average F-measure has the lowest values of all measures for all three samples. Only the macro-averaged F-measure has similar values. Other measures have been impacted too much by the dominating classes and provided much higher overall scores. Sample 1 is a great example of a classifier focusing on the dominating classes. Only the plain average F-measure and the macro-averaged F-measure are reflecting the poor classification quality of the minor

classes. Furthermore, the comparison between Sample 2 and Sample 3 emphasizes why the micro-averaged F-measure, weighted average F-measure, and accuracy are not considered in this paper. Both samples have an identical distribution of events and similar F-measure values, but in the case of Sample 2 the classifier was unable to classify the dissolving event. Yet, the micro-averaged F-measure, weighted average F-measure, and accuracy measures have much higher values in the case of Sample 2 than in the case of Sample 3, ignoring the missing classification. On the other hand, the plain average F-measure and the macro-averaged F-measure values indicate the unsuccessful classification of the dissolving event and have lower values in the case of Sample 2. Since the plain average F-measure value is easier to compute and understand than the macro-averaged F-measure, in this thesis, the plain average F-measure is used to represent the general classification quality.

However, any measure can be used to determine the classification performance, as long as it is appropriate to the problem the one is trying to solve.

Table K. The example values of different classification performance measures showing that the average F-measure and the macro-averaged F-measure best represent the general prediction quality. Other measures have been impacted too much by the dominating classes. Sample 1 was obtained from the Digg data set, while Sample 2 and Sample 3 were obtained from the MIT data set.

		Sample 1	Sample 2	Sample 3
Distribution	Continuing	6391	62	62
	Dissolving	64	9	9
	Growing	5512	78	78
	Merging	504	29	29
	Shrinking	4272	90	90
	Splitting	235	38	38
	Sum	16978	306	306
F-measure	Continuing	0,530	0,359	0,325
	Dissolving	0.029	0.000	0.235
	Growing	0.383	0.390	0.340
	Merging	0.014	0.286	0.277
	Shrinking	0.473	0.491	0.434
	Splitting	0.015	0.725	0.709
Average F-measure		0.2406	0.3752	0.3867
Macro-avg F-measure		0.2420	0.3757	0.3876
Micro-avg F-measure		0.4430	0.4379	0.4020
Weighted avg F-measure		0.4435	0.4339	0.4014
Accuracy		0.4564	0.4379	0.4020

The formulas for individual quality measures are as follows:

$$precision = \frac{tp}{tp + fp} \tag{2}$$

where tp is the number of true positive classifications and fp is the number of false positive classifications;

$$recall = \frac{tp}{tp + fn} \tag{3}$$

where fn is the number of false negative classifications;

$$accuracy = \frac{tp + tn}{tp + tn + fp + fn} \tag{4}$$

where tn is the number of true negative classifications;

$$F\text{-}measure = 2 \cdot \frac{precision \cdot recall}{precision + recall} \tag{5}$$

$$precision_{micro} = \frac{tp_1 + \cdots + tp_n}{tp_1 + fp_1 + \cdots + tp_n + fp_n} \tag{6}$$

$$recall_{micro} = \frac{tp_1 + \cdots + tp_n}{tp_1 + fn_1 + \cdots + tp_n + fn_n} \tag{7}$$

$$F\text{-}measure_{micro} = 2 \cdot \frac{precision_{micro} \cdot recall_{micro}}{precision_{micro} + recall_{micro}} \tag{8}$$

$$precision_{macro} = \frac{precision_1 + \cdots + precision_n}{n} \tag{9}$$

$$recall_{macro} = \frac{recall_1 + \cdots + recall_n}{n} \tag{10}$$

$$F\text{-}measure_{macro} = 2 \cdot \frac{precision_{macro} \cdot recall_{macro}}{precision_{macro} + recall_{macro}} \tag{11}$$

Predictive features

The list of all features considered in the paper is provided in Tab. L. The new features proposed and analyzed in this paper are highlighted in bold.

Group	Name	Description
Nodes - microscopic local	**sum_group_degree_in**	The sum of indegree [47] of nodes belonging to the community calculated within the community. Indegree is a node measure defining the number of connections directed to the node.
	**avg_group_degree_in**	The average value of indegree of nodes belonging to the community calculated within the community.
	**min_group_degree_in**	The minimum value of indegree of nodes belonging to the community calculated within the community.
	**max_group_degree_in**	The maximum value of indegree of nodes belonging to the community calculated within the community.
	**sum_group_degree_out**	The sum of outdegree [47] of nodes belonging to the community calculated within the community. Outdegree is a node measure determining the number of connections outgoing from the node.
	**avg_group_degree_out**	The average value of outdegree of nodes belonging to the community calculated within the community.
	**min_group_degree_out**	The minimum value of outdegree of nodes belonging to the community calculated within the community.
	**max_group_degree_out**	The maximum value of outdegree of nodes belonging to the community calculated within the community.
	**sum_group_degree_total**	The sum of total degree of nodes belonging to the community calculated within the community. Total degree is the sum of indegree and outdegree.
	**avg_group_degree_total**	The average value of total degree of nodes belonging to the community calculated within the community.
	**min_group_degree_total**	The minimum value of total degree of nodes belonging to the community calculated within the community.
	**max_group_degree_total**	The maximum value of total degree of nodes belonging to the community calculated within the community.

	**sum_group_betweenness**	The sum of betweenness [47] of nodes belonging to the community calculated within the community. Betweenness is a node measure describing the number of the shortest paths from all nodes to all others that pass through that node.
	**avg_group_betweenness**	The average value of betweenness of nodes belonging to the community calculated within the community.
	**min_group_betweenness**	The minimum value of betweenness of nodes belonging to the community calculated within the community.
	**max_group_betweenness**	The maximum value of betweenness of nodes belonging to the community calculated within the community.
	**sum_group_closeness**	The sum of closeness [47] of nodes belonging to the community calculated within the community. Closeness is a node measure defined as the inverse of the farness, which in turn, is the sum of distances to all other nodes.
	**avg_group_closeness**	The average value of closeness of nodes belonging to the community calculated within the community.
	**min_group_closeness**	The minimum value of closeness of nodes belonging to the community calculated within the community.
	**max_group_closeness**	The maximum value of closeness of nodes belonging to the community calculated within the community.
	**sum_group_eigenvector**	The sum of eigenvector [48] of nodes belonging to the community calculated within the community. Eigenvector is a node measure indicating the influence of a node in the network.
	**avg_group_eigenvector**	The average value of eigenvector of nodes belonging to the community calculated within the community.
	**min_group_eigenvector**	The minimum value of eigenvector of nodes belonging to the community calculated within the community.
	**max_group_eigenvector**	The maximum value of eigenvector of nodes belonging to the community calculated within the community.
	**avg_group_eccentricity**	The average value of eccentricity [49] of nodes belonging to the community calculated within the community. Eccentricity of a node is its shortest path distance from the farthest other node in the graph.
	**min_group_eccentricity**	The minimum value of eccentricity of nodes belonging to the community calculated within the community. Also called the groups diameter.
	**max_group_eccentricity**	The maximum value of eccentricity of nodes belonging to the community calculated within the community.
	avg_group_clustering_coefficient	The average local clustering coefficients of all the nodes in the community [50].
Nodes - microscopic global	**sum_network_degree_in**	The sum of indegree of nodes belonging to the community calculated within the network.
	**avg_network_degree_in**	The average value of indegree of nodes belonging to the community calculated within the network.
	**min_network_degree_in**	The minimum value of indegree of nodes belonging to the community calculated within the network.
	**max_network_degree_in**	The maximum value of indegree of nodes belonging to the community calculated within the network.
	**sum_network_degree_out**	The sum of outdegree of nodes belonging to the community calculated within the network.
	**avg_network_degree_out**	The average value of outdegree of nodes belonging to the community calculated within the network.
	**min_network_degree_out**	The minimum value of outdegree of nodes belonging to the community calculated within the network.

	**max_network_degree_out**	The maximum value of outdegree of nodes belonging to the community calculated within the network.
	**sum_network_degree_total**	The sum of total degree of nodes belonging to the community calculated within the network.
	**avg_network_degree_total**	The average value of total degree of nodes belonging to the community calculated within the network.
	**min_network_degree_total**	The minimum value of total degree of nodes belonging to the community calculated within the network.
	**max_network_degree_total**	The maximum value of total degree of nodes belonging to the community calculated within the network.
	**sum_network_betweenness**	The sum of betweenness of nodes belonging to the community calculated within the network.
	**avg_network_betweenness**	The average value of betweenness of nodes belonging to the community calculated within the network.
	**min_network_betweenness**	The minimum value of betweenness of nodes belonging to the community calculated within the network.
	**max_network_betweenness**	The maximum value of betweenness of nodes belonging to the community calculated within the network.
	**sum_network_closeness**	The sum of closeness of nodes belonging to the community calculated within the network.
	**avg_network_closeness**	The average value of closeness of nodes belonging to the community calculated within the network.
	**min_network_closeness**	The minimum value of closeness of nodes belonging to the community calculated within the network.
	**max_network_closeness**	The maximum value of closeness of nodes belonging to the community calculated within the network.
	**sum_network_eigenvector**	The sum of eigenvector of nodes belonging to the community calculated within the network.
	**avg_network_eigenvector**	The average value of eigenvector of nodes belonging to the community calculated within the network.
	**min_network_eigenvector**	The minimum value of eigenvector of nodes belonging to the community calculated within the network.
	**max_network_eigenvector**	The maximum value of eigenvector of nodes belonging to the community calculated within the network.
	avg_network_clustering_coefficient	The average of the local clustering coefficients of all the nodes in the network [50].
Group - mesoscopic	group_size	The number of nodes in the group.
	group_density	The number of connections between nodes in the group in relation to all possible connections between them [50].
	group_cohesion	The vertex connectivity of the community [51].
	group_coefficient_global	The ratio of the triangles and the connected triples in the community [50].
	**group_reciprocity**	A fraction of edges that are reciprocated within the community [52].
	**group_leadership**	A measure describing centralization in the community (the largest value is for a star network) [47].
	**neighborhood_out**	The number of nodes outside the community that have incoming connection from the nodes inside the community divided by the number of nodes in the community.
	**neighborhood_in**	The number of nodes outside the community that have outgoing connection to the nodes inside the community divided by the number of nodes in the community.

	**neighborhood_all**	The number of nodes outside the community that are connected to the nodes inside the community divided by the number of nodes in the community.
	**group_adhesion**	The minimum number of edges needed to be removed to obtain a community which is not strongly connected [51].
	**alpha**	The GED inclusion measure of group G_i from time window T_n in group G_j from T_{n+1} [53].
	**beta**	The GED inclusion measure of group G_j from time window T_{n+1} in group G_i from T_n [53].
	network_ratio_size	The ratio of group_size to network_size.
	network_ratio_density	The ratio of group_density to network_density.
	network_ratio_cohesion	The ratio of group_cohesion to network_cohesion.
	**network_ratio_coefficient_global**	The ratio of group_coefficient_global to network_coefficient_global.
	**network_ratio_coefficient_average**	The ratio of group_clustering_coefficient to network_clustering_coefficient.
	**network_ratio_reciprocity**	The ratio of group_reciprocity to network_reciprocity.
	**network_ratio_leadership**	The ratio of group_leadership to network_leadership.
	**network_ratio_eccentricity**	The ratio of avg_group_eccentricity to network_avg_eccentricity.
	**network_ratio_adhesion**	The ratio of group_adhesion to network_adhesion.
Network - macroscopic	network_size	The number of nodes in the network.
	network_density	The number of connections between nodes in the network in relation to all possible connections between them.
	network_cohesion	The vertex connectivity of the network.
	network_coefficient_global	The ratio of the triangles and the connected triples in the network.
	network_coefficient_average	The average of the local clustering coefficients of all the nodes in the network.
	**network_reciprocity**	A fraction of edges that are reciprocated within the network.
	**network_leadership**	A measure describing centralization in the network (the largest value is for a star network).
	**network_avg_eccentricity**	The average value of eccentricity of nodes within the network.
	**network_adhesion**	The minimum number of edges needed to be removed to obtain a graph which is not strongly connected.

Table L. Predictive features - newly proposed features (bolded) and features known from the literature.

GED

The GED method uses the sizes and inclusion measures of two groups in the consecutive time frames to identify the event type. The alpha and beta parameters can be adjusted according to the needs. For example, to keep only evolutions between very similar groups the values of alpha and beta should be kept high, e.g., at the level of 80%). On the other hand, sometimes the network evolves very rapidly, and the only way to track some evolutions is to lower the alpha and beta parameters e.g. to 30%. Tab. M demonstrates the influence of the alpha and beta values on the number of identified events of the particular type for the IrvinaMessages data set.

alpha	beta	forming	dissolving	shrinking	growing	continuing	splitting	merging	total
10	10	362	350	424	351	97	217	164	1965
10	20	362	350	403	291	97	82	222	1807
10	30	362	350	378	280	93	66	233	1762
10	40	362	350	370	269	93	42	244	1730
10	50	362	350	347	260	94	35	253	1701
10	60	362	350	334	259	94	35	254	1688

10	70	362	350	322	258	94	38	255	1679
10	80	362	350	319	257	94	37	256	1675
10	90	362	350	319	257	94	37	256	1675
10	100	362	350	319	257	94	37	256	1675
20	10	362	350	360	323	91	280	70	1836
20	20	362	350	349	280	87	120	89	1637
20	30	362	350	327	272	84	60	97	1552
20	40	362	350	300	267	84	37	102	1502
20	50	362	350	273	262	86	17	107	1457
20	60	362	350	249	261	86	7	108	1423
20	70	362	350	231	260	86	6	109	1404
20	80	362	350	229	260	86	2	109	1398
20	90	362	350	229	260	86	2	109	1398
20	100	362	350	229	260	86	2	109	1398
30	10	362	350	349	297	88	294	60	1800
30	20	362	350	342	261	78	129	40	1562
30	30	362	350	318	253	72	58	35	1448
30	40	362	350	278	251	71	40	37	1389
30	50	362	350	242	247	73	14	41	1329
30	60	362	350	205	247	73	7	41	1285
30	70	362	350	181	246	73	6	42	1260
30	80	362	350	175	246	73	2	42	1250
30	90	362	350	175	246	73	2	42	1250
30	100	362	350	175	246	73	2	42	1250
40	10	362	350	339	289	84	308	48	1780
40	20	362	350	337	248	75	137	22	1531
40	30	362	350	315	228	69	61	16	1401
40	40	362	350	270	221	64	39	18	1324
40	50	362	350	229	218	64	13	21	1257
40	60	362	350	185	218	64	5	21	1205
40	70	362	350	151	218	64	4	21	1170
40	80	362	350	143	218	64	0	21	1158
40	90	362	350	140	218	64	0	21	1155
40	100	362	350	140	218	64	0	21	1155
50	10	362	350	345	280	83	303	52	1775
50	20	362	350	339	224	74	136	19	1504
50	30	362	350	315	191	67	62	16	1363
50	40	362	350	270	179	61	38	15	1275
50	50	362	350	223	175	52	13	16	1191
50	60	362	350	172	175	52	5	16	1132
50	70	362	350	131	175	52	4	16	1090
50	80	362	350	116	175	52	0	16	1071
50	90	362	350	110	175	52	0	16	1065
50	100	362	350	110	175	52	0	16	1065
60	10	362	350	343	272	83	305	43	1758
60	20	362	350	337	208	74	138	13	1482
60	30	362	350	314	166	67	63	8	1330
60	40	362	350	269	142	60	39	9	1231
60	50	362	350	222	134	50	14	11	1143
60	60	362	350	169	136	47	6	8	1078
60	70	362	350	129	136	46	2	8	1033
60	80	362	350	108	136	46	0	8	1010

60	90	362	350	101	136	46	0	8	1003
60	100	362	350	101	136	46	0	8	1003
70	10	362	350	343	265	83	305	38	1746
70	20	362	350	337	193	74	138	7	1461
70	30	362	350	314	146	67	63	4	1306
70	40	362	350	269	113	60	39	6	1199
70	50	362	350	222	100	50	14	6	1104
70	60	362	350	169	101	47	6	4	1039
70	70	362	350	128	101	44	2	4	991
70	80	362	350	106	101	43	0	4	966
70	90	362	350	99	101	43	0	4	959
70	100	362	350	99	101	43	0	4	959
80	10	362	350	341	262	83	307	39	1744
80	20	362	350	337	188	74	138	7	1456
80	30	362	350	314	138	67	63	5	1299
80	40	362	350	269	105	60	39	4	1189
80	50	362	350	222	90	50	14	4	1092
80	60	362	350	169	88	47	6	2	1024
80	70	362	350	128	87	42	2	2	973
80	80	362	350	106	87	35	0	2	942
80	90	362	350	99	87	35	0	2	935
80	100	362	350	99	87	35	0	2	935
90	10	362	350	341	261	83	307	40	1744
90	20	362	350	337	187	74	138	8	1456
90	30	362	350	314	137	67	63	6	1299
90	40	362	350	269	103	60	39	2	1185
90	50	362	350	222	86	50	14	2	1086
90	60	362	350	169	83	47	6	0	1017
90	70	362	350	128	81	42	2	0	965
90	80	362	350	106	79	35	0	0	932
90	90	362	350	99	79	35	0	0	925
90	100	362	350	99	79	35	0	0	925
100	10	362	350	341	261	83	307	40	1744
100	20	362	350	337	187	74	138	8	1456
100	30	362	350	314	137	67	63	6	1299
100	40	362	350	269	103	60	39	2	1185
100	50	362	350	222	86	50	14	2	1086
100	60	362	350	169	83	47	6	0	1017
100	70	362	350	128	80	42	2	0	964
100	80	362	350	106	78	35	0	0	931
100	90	362	350	99	78	35	0	0	924
100	100	362	350	99	78	35	0	0	924

Table M. The number of events of the particular type tracked with the GED method for different values of the alpha and beta parameters for the IrvinaMessages data set.

Acknowledgements

This work was partially supported by The Polish National Science Centre, the projects no. 2016/21/B/ST6/01463 and 2016/21/D/ST6/02408 and by the European Union's Horizon 2020 research and innovation programme under the Marie Skłodowska-Curie grant agreement No. 691152 (RENOIR) and the Polish Ministry of Science and Higher

Education fund for supporting internationally co-financed projects in 2016-2019, agreement no. 3628/H2020/2016/2. This research was supported in part by PLGrid Infrastructure.

References

1. Ilhan, N. & Oguducu, I. G. Community event prediction in dynamic social networks. In *Proceedings of the 12th International Conference on Machine Learning and Applications (ICMLA'2013)*, vol. 1, 191–196 (IEEE, 2013).

2. İlhan, N. & Öğüdücü, Ş. G. Feature identification for predicting community evolution in dynamic social networks. *Engineering Applications of Artificial Intelligence* **55**, 202–218 (2016).

3. Takaffoli, M., Rabbany, R. & Zaïane, O. R. Community evolution prediction in dynamic social networks. In *IEEE/ACM International Conference on Advances in Social Networks Analysis and Mining (ASONAM'2014)*, 9–16 (IEEE, 2014).

4. Diakidis, G., Karna, D., Fasarakis-Hilliard, D., Vogiatzis, D. & Paliouras, G. Predicting the evolution of communities in social networks. In *Proceedings of the 5th International Conference on Web Intelligence, Mining and Semantics (WIMS'15)*, 1 (ACM, 2015).

5. Goldberg, M., Magdon-Ismail, M., Nambirajan, S. & Thompson, J. Tracking and predicting evolution of social communities. In *Proceedings of the 2011 IEEE Third International Conference on Privacy, Security, Risk and Trust (PASSAT) and 2011 IEEE Third Inernational Conference on Social Computing (SocialCom)*, 780–783 (IEEE, 2011).

6. Goldberg, M., Magdon-Ismail, M. & Thompson, J. Identifying long lived social communities using structural properties. In *Proceedings of the 2012 International Conference on Advances in Social Networks Analysis and Mining (ASONAM 2012)*, 647–653 (IEEE Computer Society, 2012).

7. Kairam, S. R., Wang, D. J. & Leskovec, J. The life and death of online groups: Predicting group growth and longevity. In *Proceedings of the Fifth ACM International Conference on Web Search and Data Mining (WSDM'12)*, 673–682 (ACM, 2012).

8. Ley, M. The dblp computer science bibliography: Evolution, research issues, perspectives. In *String processing and information retrieval*, 481–486 (Springer, 2002).

9. De Choudhury, M., Sundaram, H., John, A. & Seligmann, D. D. Social synchrony: Predicting mimicry of user actions in online social media. In *International Conference on Computational Science and Engineering CSE'09.*, vol. 4, 151–158 (IEEE, 2009).

10. Viswanath, B., Mislove, A., Cha, M. & Gummadi, K. P. On the evolution of user interaction in facebook. In *Proceedings of the 2nd ACM workshop on Online social networks*, 37–42 (ACM, 2009).

11. Isella, L. *et al.* What's in a crowd? Analysis of face-to-face behavioral networks. *Journal of theoretical biology* **271**, 166–180 (2011).

12. Opsahl, T. & Panzarasa, P. Clustering in weighted networks. *Social networks* **31**, 155–163 (2009).

13. Rossi, R. & Ahmed, N. The network data repository with interactive graph analytics and visualization. In *AAAI*, vol. 15, 4292–4293 (2015).

14. Prosper loans network dataset – konect, april 2017. (2017). URL `http://konect.uni-koblenz.de/networks/prosper-loans`.

15. Eagle, N. & Pentland, A. S. Reality mining: sensing complex social systems. *Personal and ubiquitous computing* **10**, 255–268 (2006).

16. Gómez, V., Kaltenbrunner, A. & López, V. Statistical analysis of the social network and discussion threads in slashdot. In *Proceedings of the 17th international conference on World Wide Web*, 645–654 (ACM, 2008).

17. Conover, M. *et al.* Political polarization on twitter. *ICWSM* **133**, 89–96 (2011).

18. Guyon, I. & Elisseeff, A. An introduction to variable and feature selection. *Journal of machine learning research* **3**, 1157–1182 (2003).

19. Bäck, T., Fogel, D. B. & Michalewicz, Z. *Evolutionary computation 1: Basic algorithms and operators*, vol. 1 (CRC press, 2000).

20. Alpaydm, E. Combined 5× 2 cv F test for comparing supervised classification learning algorithms. *Neural computation* **11**, 1885–1892 (1999).

21. Yang, J. & Honavar, V. Feature subset selection using a genetic algorithm. *IEEE Intelligent Systems and their Applications* **13**, 44–49 (1998).

22. Pedregosa, F. *et al.* Scikit-learn: Machine learning in Python. *Journal of Machine Learning Research* **12**, 2825–2830 (2011).

23. Fortin, F.-A., De Rainville, F.-M., Gardner, M.-A., Parizeau, M. & Gagné, C. DEAP: Evolutionary algorithms made easy. *Journal of Machine Learning Research* **13**, 2171–2175 (2012).

24. Saganowski, S. Replication data for: Analysis of group evolution prediction in complex networks (2018). URL `http://dx.doi.org/10.7910/DVN/ONOFS7`. DOI 10.7910/DVN/ONOFS7.

25. Hall, M. *et al.* The weka data mining software: an update. *ACM SIGKDD explorations newsletter* **11**, 10–18 (2009).

26. Cohen, W. W. Fast effective rule induction. In *Machine Learning Proceedings 1995*, 115–123 (Elsevier, 1995).

27. Kohavi, R. The power of decision tables. In *European conference on machine learning*, 174–189 (Springer, 1995).

28. Chang, C.-C. & Lin, C.-J. Libsvm: a library for support vector machines. *ACM transactions on intelligent systems and technology (TIST)* **2**, 27 (2011).

29. Breiman, L. Random forests. *Machine learning* **45**, 5–32 (2001).

30. Quinlan, J. R. *C4. 5: programs for machine learning* (Elsevier, 2014).

31. Breiman, L., Friedman, J., Stone, C. J. & Olshen, R. A. *Classification and regression trees* (CRC press, 1984).

32. John, G. H. & Langley, P. Estimating continuous distributions in bayesian classifiers. In *Proceedings of the Eleventh conference on Uncertainty in artificial intelligence*, 338–345 (Morgan Kaufmann Publishers Inc., 1995).

33. Aha, D. W., Kibler, D. & Albert, M. K. Instance-based learning algorithms. *Machine learning* **6**, 37–66 (1991).

34. Cleary, J. G. & Trigg, L. E. K*: An instance-based learner using an entropic distance measure. In *Machine Learning Proceedings 1995*, 108–114 (Elsevier, 1995).

35. Freund, Y., Schapire, R. E. *et al.* Experiments with a new boosting algorithm. In *Icml*, vol. 96, 148–156 (Bari, Italy, 1996).

36. Iba, W. & Langley, P. Induction of one-level decision trees. In *Machine Learning Proceedings 1992*, 233–240 (Elsevier, 1992).

37. Breiman, L. Bagging predictors. *Machine learning* **24**, 123–140 (1996).

38. Le Cessie, S. & Van Houwelingen, J. C. Ridge estimators in logistic regression. *Applied statistics* 191–201 (1992).

39. Friedman, M. The use of ranks to avoid the assumption of normality implicit in the analysis of variance. *Journal of the american statistical association* **32**, 675–701 (1937).

40. Shaffer, J. P. Modified sequentially rejective multiple test procedures. *Journal of the American Statistical Association* **81**, 826–831 (1986).

41. Alcalá-Fdez, J. *et al.* Keel: a software tool to assess evolutionary algorithms for data mining problems. *Soft Computing* **13**, 307–318 (2009).

42. Cerri, R., Pappa, G. L., Carvalho, A. C. P. & Freitas, A. A. An extensive evaluation of decision tree–based hierarchical multilabel classification methods and performance measures. *Computational Intelligence* **31**, 1–46 (2015).

43. Ferri, C., Hernández-Orallo, J. & Modroiu, R. An experimental comparison of performance measures for classification. *Pattern Recognition Letters* **30**, 27–38 (2009).

44. Sokolova, M. & Lapalme, G. A systematic analysis of performance measures for classification tasks. *Information Processing & Management* **45**, 427–437 (2009).

45. Huang, J. & Ling, C. X. Constructing new and better evaluation measures for machine learning. In *IJCAI*, 859–864 (2007).

46. Yang, Y. An evaluation of statistical approaches to text categorization. *Information retrieval* **1**, 69–90 (1999).

47. Freeman, L. C. Centrality in social networks conceptual clarification. *Social networks* **1**, 215–239 (1978).

48. Bonacich, P. Factoring and weighting approaches to status scores and clique identification. *Journal of mathematical sociology* **2**, 113–120 (1972).

49. Harary, F. Graph theory. 1969.

50. Wasserman, S. & Faust, K. *Social network analysis: Methods and applications*, vol. 8 (Cambridge university press, 1994).

51. White, D. R. & Harary, F. The cohesiveness of blocks in social networks: Node connectivity and conditional density. *Sociological Methodology* **31**, 305–359 (2001).

52. Newman, M. *Networks: an introduction* (Oxford university press, 2010).

53. Bródka, P., Saganowski, S. & Kazienko, P. GED: the method for group evolution discovery in social networks. *Social Network Analysis and Mining* **3**, 1–14 (2013).
